# Maternal bile acid transporter deficiency promotes neonatal demise

**DOI:** 10.1038/ncomms9186

**Published:** 2015-09-29

**Authors:** Yuanyuan Zhang, Fei Li, Yao Wang, Aaron Pitre, Zhong-ze Fang, Matthew W. Frank, Christopher Calabrese, Kristopher W. Krausz, Geoffrey Neale, Sharon Frase, Peter Vogel, Charles O. Rock, Frank J. Gonzalez, John D. Schuetz

**Affiliations:** 1 Department of Pharmaceutical Sciences, St Jude Children's Research Hospital, 262 Danny Thomas Place, Memphis, Tennessee 38105, USA; 2 Laboratory of Metabolism, National Cancer Institute, National Institutes of Health, Bethesda, Maryland 20892, USA; 3 Department of Infectious Diseases, St Jude Children's Research Hospital, 262 Danny Thomas Place, Memphis, Tennessee 38105, USA; 4 Small Animal Imaging Core, St Jude Children's Research Hospital, 262 Danny Thomas Place, Memphis, Tennessee 38105, USA; 5 Hartwell Center, St Jude Children's Research Hospital, 262 Danny Thomas Place, Memphis, Tennessee 38105, USA; 6 Cellular Imaging Shared Resource, St Jude Children's Research Hospital, 262 Danny Thomas Place, Memphis, Tennessee 38105, USA; 7 Department of Pathology, St Jude Children's Research Hospital, 262 Danny Thomas Place, Memphis, Tennessee 38105, USA

## Abstract

Intrahepatic cholestasis of pregnancy (ICP) is associated with adverse neonatal survival and is estimated to impact between 0.4 and 5% of pregnancies worldwide. Here we show that maternal cholestasis (due to Abcb11 deficiency) produces neonatal death among all offspring within 24 h of birth due to atelectasis-producing pulmonary hypoxia, which recapitulates the neonatal respiratory distress of human ICP. Neonates of Abcb11-deficient mothers have elevated pulmonary bile acids and altered pulmonary surfactant structure. Maternal absence of Nr1i2 superimposed on Abcb11 deficiency strongly reduces maternal serum bile acid concentrations and increases neonatal survival. We identify pulmonary bile acids as a key factor in the disruption of the structure of pulmonary surfactant in neonates of ICP. These findings have important implications for neonatal respiratory failure, especially when maternal bile acids are elevated during pregnancy, and highlight potential pathways and targets amenable to therapeutic intervention to ameliorate this condition.

## Abstract (web summary)

The mechanisms underlying perinatal mortality due to intrahepatic cholestasis of pregnancy are not fully understood. Here, the authors show that absence of the nuclear receptor and bile acid regulator Nrli2 and the biliary transporter Abcb11 strongly reduces maternal serum bile acid levels, improving neonatal survival.

## Graphical abstract

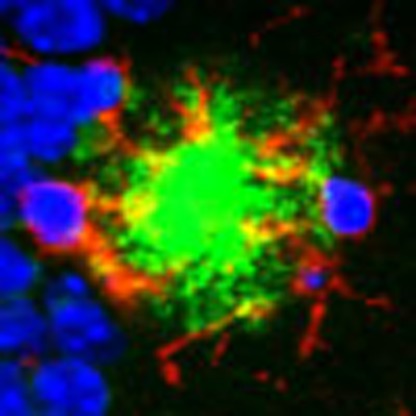

Some women, with no history of liver disease, develop cholestasis during pregnancy that typically presents late in gestation. These women are diagnosed with intrahepatic cholestasis of pregnancy (ICP) and exhibit pruritus and elevated serum bile acids. The frequency of ICP varies between 0.4 and 5% (refs 1, 2, 3, 4, 5, 6) with the prevalence being higher in some ethnic groups. These findings and evidence of family clustering suggests a genetic basis with a number of biliary transporters (for example, *ABCB4* (*MDR3*), *ABCB11* (*BSEP*), *ABCC2* and *ATP8B1*) being implicated as causing ICP. In addition, environmental factors might contribute, as inhibition of these transporters by medications or dietary constituents can also elevate maternal bile acids^7^. For example, drug-induced inhibition of the primary hepatic bile acid transporter, ABCB11, produces cholestasis in man and rodents^7^. Nonetheless, the maternal symptoms of ICP resolve within a short period of time postpartum.

In contrast to the benign outcome of maternal ICP, neonatal outcomes of ICP are adverse^1^,^8^,^9^. In particular, respiratory distress is unfavourable. The mechanism(s) accounting for ICP-associated infant perinatal mortality, due to respiratory distress, are unclear^1^,^2^,^10^. Respiratory distress is an unexpected outcome for term and near-term infants. It is possible that elevated maternal bile acids impact lung development, however, while possible, this seems unlikely given the apparently normal lung maturation^10^. Other studies have suggested that lung surfactant levels are depleted^10^.

We previously demonstrated that *Abcb11*^−/−^ mice develop intrahepatic cholestasis^11^. The goal of this work was to determine if pregnant *Abcb11*^*−/−*^ mice display neonatal phenotypes similar to human ICP. Our studies showed neonates from *Abcb11*^−/−^ mothers exhibited perinatal lethality. The basis for the survival defect was related to pulmonary failure secondary to defects in surfactant structure associated with pulmonary bile acid accumulation. We then determined that bile acids disrupt the structural order of pulmonary surfactant lipids. Importantly, when mothers lack the nuclear receptor Nr1i2 (*aka Pxr*, a regulator of bile acid homoeostasis) and Abcb11, maternal bile acids were reduced and neonatal survival was dramatically increased. These findings not only represent an important animal model to advance treatment and understanding of ICP, they also reveal pathways that might be exploited to improve neonatal outcomes.

## Results

### Newborn death caused by atelectasis

**Figure 1:**
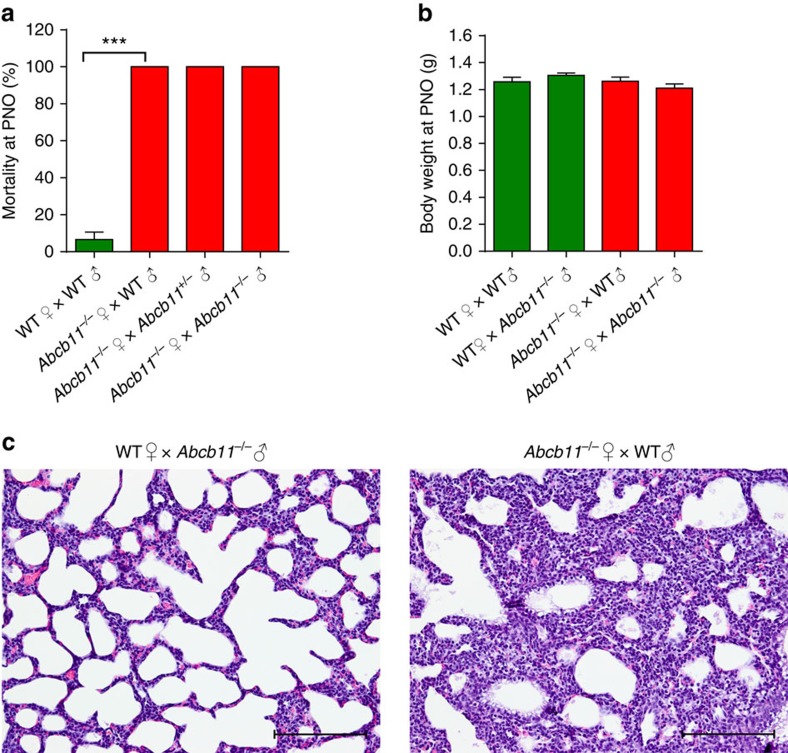
Maternal *Abcb11* deficiency produces neonatal lethality. (**a**) *Abcb11*^−/−^ females were interbred with WT (*n*=2), *Abcb11*^+/−^ and *Abcb11*^−/−^ males (*n*=4). No neonates survived more than 24 h. Green bars indicate WT mother, while red bars indicate an *Abcb11*^−/−^ mother. (**b**) BW of neonates at PN0 reveals no significant difference regardless of parentage. WT♀ × WT♂, *n*=14; WT♀ × *Abcb11*^−/−^♂, *n*=21, *Abcb11*^*−/−*^♀ × WT♂, *n*=18; *Abcb11*^*−/−*^♀ × *Abcb11*^*−/−*^♂, *n*=9. (**c**) Hematoxylin and eosin stain reveals collapsed alveoli from the lungs of neonates carried by *Abcb11*^−/−^ mothers. All experimental values are presented as mean±s.e.m. The height of the error bar=1 standard error. Statistical analysis was performed using two-tailed Student's *t*-test, ****P<0.0001. Scale bar, 200 μm.

We discovered, by serendipity, that neonatal pups produced by *Abcb11*^−/−^ mothers did not survive. By further interbreeding studies, we found 100% of newborns die within the first 24 h from *Abcb11*^−/−^ mothers, despite the different genotypes of fathers (wild type (WT), *Abcb11*^−/+^ and *Abcb11*^−/−^; Fig. 1a). These interbreeding schemes demonstrated that neither the father's nor neonate's *Abcb11* genotype were essential contributors to neonatal death. Although the body weight (BW) of neonates from *Abcb11*^−/−^ mothers was similar to those of WT (Fig. 1b), the mice were morphologically indistinguishable (Supplementary Fig. 1). However, a consistent and major phenotype distinguishing neonates produced by *Abcb11*^−/−^ mothers was their pale pink colour compared with the normal ruddy, reddish-pink of healthy neonatal pups (Supplementary Fig. 1). This observation suggested that neonatal death might be due to poor oxygenation.

To rule out neonatal genotype as the cause of postnatal death, parental mice were intercrossed to produce only heterozygous offspring as follows: *Abcb11*^−/−^ males were bred with WT females or *Abcb11*^−/−^ females bred with WT males. Virgin *Abcb11*^−/−^ females have been shown to elevate serum bile acid levels and develop cholestasis-induced liver injuries^11^ and the studies conducted herein used mice ≤3 months to minimize liver injury. Necropsy of these heterozygous neonates of *Abcb11*^−/−^ females revealed normal morphology and histology in the major organs, including liver, kidney, brain and heart. The only exception was the collapsed alveoli (atelectasis) in the lungs of the neonates from *Abcb11*^−/−^ mothers (Fig. 1c).

Interestingly, atelectasis appears unrelated to altered lung development or vasculature defects. Using microarray analysis and timed pregnancy, we evaluated expression of developmental genes at embryonic day 18.5 (E18.5) and postnatal day 0 (PN0; Supplementary Fig. 2a). Expression of key genes in lung development was unaltered between the neonates produced by WT and *Abcb11*^−/−^ mothers. Further, there was no evidence for changes in expression of genes related to lipid metabolism (Supplementary Fig. 2b). Using *Aqp5* and *Meca32*, as markers of type I pneumocytes and vascular cells, respectively, we found that protein (Supplementary Fig. 3a) or mRNA expression (Supplementary Fig. 3b) were no different in the postnatal lungs (PN0, the day of birth) between neonates from *Abcb11*^−/−^ mothers interbred with WT males, WT females intercrossed with WT males and WT females mated to *Abcb11*^−/−^ males. We also evaluated changes in proliferation for the lungs. Ki67 staining, an indicator of proliferation, showed extensive, but similar proliferation in lungs of neonates from either WT or *Abcb11*^−/−^ mothers (Supplementary Fig. 4a). There was little detectable apoptosis as measured by Caspase 3-positive cells, in neonatal lungs and no detectable difference between neonates from *Abcb11*^−/−^ versus WT mothers (Supplementary Fig. 4b).

Maternal corticosterone rises during the last trimester of pregnancy and is crucial for normal lung development and surfactant gene expression^12^,^13^. Maternal serum corticosterone concentrations were measured before mating (P0) and at day 17.5 of pregnancy (P17.5; Supplementary Fig. 5). As expected, the level of corticosterone increased during pregnancy in both *Abcb11*^−/−^ and WT mothers, but the levels at P0 and P17.5 were not significantly different between WT and *Abcb11*^−/−^ mothers (Supplementary Fig. 5). This indicates that the collapsed alveoli in neonates from *Abcb11*^−/−^ mothers are not due to maternal insufficiency of corticosterone. Consistently, embryonic lungs at E17.5 and E18.5 exhibited no morphological differences (Supplementary Fig. 6a) or changes in surfactant protein c, Sftpc, a marker of type II cells (Supplementary Fig. 6b). These data suggest that the collapsed alveoli are not due to impaired foetal lung development.

### Altered pulmonary surfactant induced hypoxia

**Figure 2:**
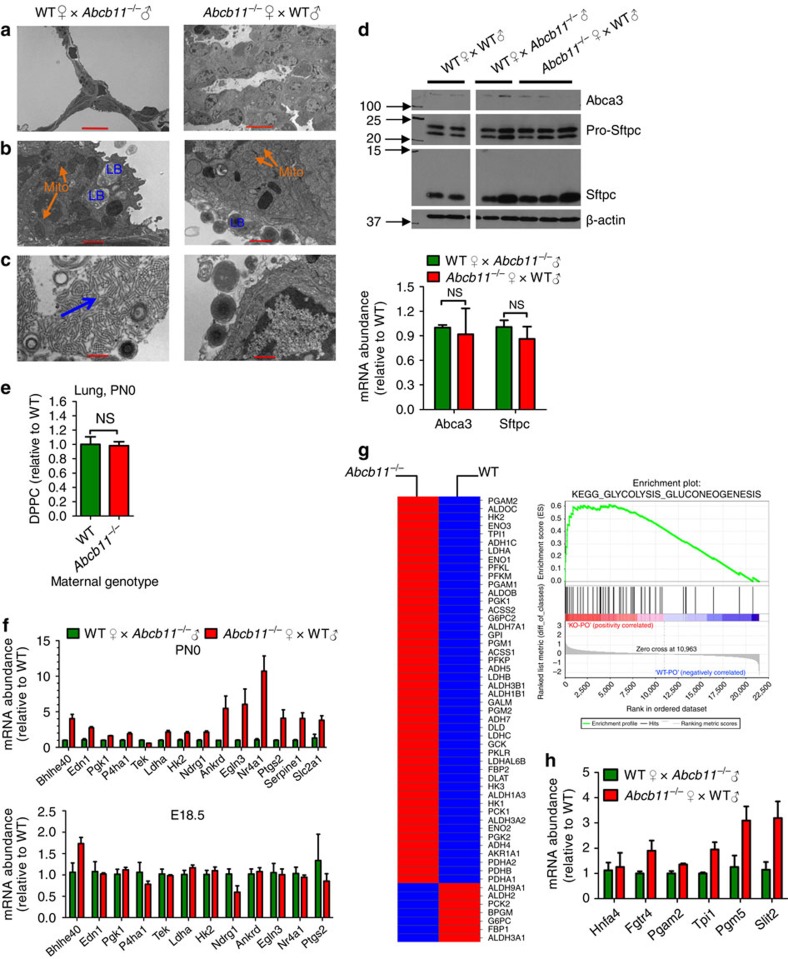
Surfactant morphology in neonates of *Abcb11*^−/−^ mothers is disrupted and associated with pulmonary hypoxia gene expression. (**a**) Transmission electron microscopy reveals thickened septa in the neonatal lungs in offspring of *Abcb11*^−/−^♀ × WT♂ compared with the thin septa in the offspring of WT♀ × *Abcb11*^−/−^♂. (**b**) The type II pneumocytes release lamellar bodies (LB) regardless of maternal genotype. Mito, mitochondria (orange arrows). (**c**) The released surfactant in neonates from WT mothers reveals an organized structure ‘tubular myelin' (blue arrow) unlike the one in neonates from *Abcb11*^−/−^ mothers. (**d**) Immunoblot analysis (upper panel) reveals no reduction in proteins important for LB and this is consistent with real-time Q-PCR (lower panel) of RNA from WT♀ × WT♂, WT♀ × *Abcb11*^−/−^♂ and *Abcb11*^−/−^♀ × WT♂. Abca3, a marker for lamellar bodies; pro-Sftpc and Sftpc, surfactant proteins. (**e**) UPLC-ESI-QTOFMS was used to determine the phospholipid composition of pulmonary surfactant in neonatal lung from offspring of the following interbreeding: WT♀ × *Abcb11*^−/−^♂ and *Abcb11*^−/−^♀ × WT♂. *n*=3. DPPC, dipalmitoylphosphatidylcholine. (**f**) Hypoxia-inducible factor target gene abundance was determined by real-time Q-PCR in the neonatal (PN0) and embryonic (E18.5) lungs of offspring from WT♀ × *Abcb11*^−/−^♂ and *Abcb11*^−/−^♀ × WT♂, indicating upregulation of hypoxia-inducible factor pathway. *n*=4. (**g**) GSEA identified upregulation of genes in the glycolysis pathway in neonates from *Abcb11*^−/−^♀ × WT♂. Red indicates increased gene expression and blue decreased gene expression. (**h**) The increased expression of selected glycolytic genes was confirmed by real-time Q-PCR. Green bars indicate WT and red bars indicate *Abcb11*^−/−^♀. All experimental values are presented as mean±s.e.m. The height of the error bar=1 standard error. Statistical analysis was processed using two-tailed Student's *t*-test. *P<0.05. Scale bars, 10μm in (**a**) and 1μm in (**b**) and (**c**).

Transmission electron microscopy revealed an abnormal ultrastructure of the lungs of neonates from *Abcb11*^−/−^ mothers. The septa between alveoli were thicker compared with the typically thin septa in the neonates of WT females (Fig. 2a). The mitochondria (labelled ‘mito') and organelles that store and secrete surfactant, and the lamellar bodies (labelled ‘LB' Fig. 2b) were readily detected in neonatal lungs from both *Abcb11*^−/−^ and WT mothers. However, the structure of the surfactant released into the lumen of the airspaces of neonates from *Abcb11*^−/−^ mothers was different, exhibiting compact coils after exocytosis. This was unlike the surfactant from neonates of WT mothers, which, on release, form ‘net-like' structures referred to as ‘tubular myelin' (highlighted by the blue arrow in Fig. 2c)^14^. While this may suggest a difference in surfactant composition, the mRNA and protein expression of *Abca3*, a gene essential for lamellar body biogenesis^15^,^16^ and surfactant protein, Pro-Sftpc, and its processed form, Sftpc, were no different among the neonatal lungs (Fig. 2d). We also investigated if the relative amounts of surfactant phospholipids differed between neonates. There was no significant difference in the amount of the major primary lung phospholipid, dipalmitoylphosphatidylcholine between the neonates from WT and *Abcb11*^−/−^ mothers (Fig. 2e). Thus, the structural differences in the released surfactant are not attributed to changes in surfactant protein or lipid composition.

To determine if the neonatal lungs from *Abcb11*^−/−^ mothers were poorly oxygenated, we isolated lungs from prenatal embryos (E18.5) and postnatal (PN0) neonates, and measured the expression level of genes associated with the hypoxia-inducible factor pathway^17^. Real-time Q-PCR indicated a strong increase in the expression of hypoxia-inducible factor target genes, but only in PN0 lungs and not in prenatal (E18.5) lungs from *Abcb11*^−/−^ mothers (Fig. 2f). Upregulation of the glycolytic pathway also occurs under hypoxia^18^ and, consistent with this, Gene Set Enrichment Analysis (GSEA) revealed that the expression of genes in the glycolytic pathway was upregulated in the lungs of neonates from *Abcb11*^−/−^ mothers (Fig. 2g). Gene changes in this pathway were confirmed by real-time Q-PCR (Fig. 2h). In total, these data demonstrate that altered pulmonary surfactant associates with poor pulmonary oxygenation and neonatal death.

### Bile acids disrupt surfactant structure

**Figure 3:**
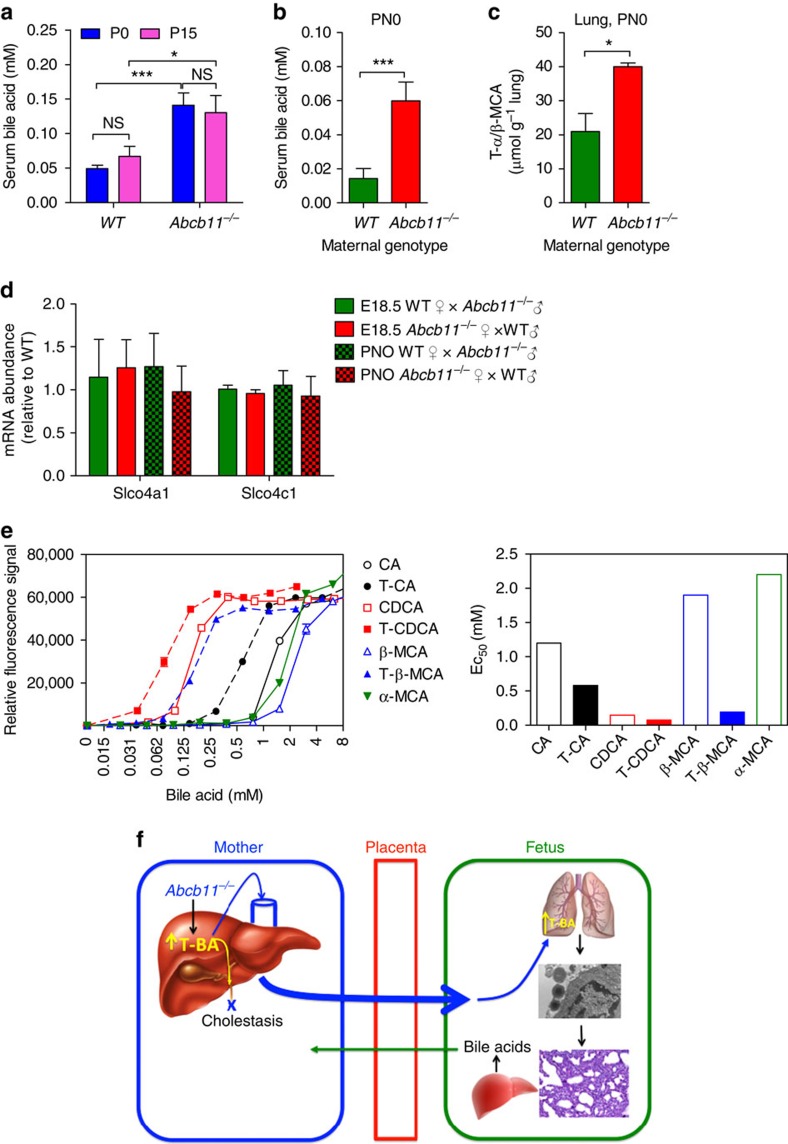
Neonates of *Abcb11*-deficient mothers have elevated bile acids in serum and lung and bile acids disrupt ‘surfactant-like' liposomes. (**a**) Total serum bile acids determined by enzymatic assay in WT and *Abcb11*^−/−^ virgins (P0, blue bar) and pregnant females (P15, purple bar). WT (P0), *n*=14; WT (P15), *n*=13; *Abcb11*^−/−^ (P0), *n*=9; *Abcb11*^*−/−*^ (P15), *n*=6. (**b**) Neonatal serum bile acid concentration was greater in neonates from *Abcb11*^−/−^♀ × WT♂ (*n*=7) intercrosses. The green bar indicates a WT maternal genotype and the red bar *Abcb11*^−/−^. (**c**) Neonatal lungs from *Abcb11*^−/−^ mothers had higher concentrations of taurine conjugated muricholic acid determined by UPLC-ESI-QTOFMS. *n*=3. The green bar indicates a WT maternal genotype and the red bar *Abcb11*^−/−^. (**d**) Messenger RNA levels of bile acid uptake transporters (Slco4a1 and Slco4c1) were determined by real-time Q-PCR from lungs of heterozygote embryos (E18.5) and neonates (PN0) of the following intercrosses: WT♀ × *Abcb11*^−/−^♂ and *Abcb11*^−/−^♀ × WT♂. *n*=4. (**e**) Large uni-lamellar liposomes were prepared from surfactant phospholipids (1-palmitoyl-2-oleoyl-*sn*-glycero-3-phosphocholine, 1,2-dipalmitoyl-*sn*-glycero-3-phosphocholine and 1,2-dioleoyl-sn-glycero-3-phospho-(1'-rac-glycerol) (5:4:1)). The liposomes were challenged with bile acids or their taurine conjugates over a range of concentrations (0.01–8.0 mM) to determine the potency to bilayer phospholipids. The dose-effect curve shown in left panel, and EC_50_ of individual bile salts in right panel. Emission filter, 355 nm; excitation filter, 530 nm. Each bile acid is colour-coded for recognition as described in the legend. CA, cholic acid; TCA, taurocholic acid; T-β-MCA, tauro-β-muricholic acid; α-MCA, α- muricholic acid. (**f**) A schematic model depicting an association of neonatal bile acids with maternal bile acid concentrations and with postnatal hypoxia. All experimental values are presented as mean±s.e.m. The height of the error bar=1 standard error. Statistical analysis was processed using two-tailed Student's *t*-test. *P<0.05; **P<0.01; ***P<0.001; ****P<0.0001.

We next investigated how maternal bile acids affect neonatal pulmonary surfactant. We found a positive association between serum bile acid concentrations in the mothers and neonates. *Abcb11*^−/−^ serum bile acid concentration was strongly elevated during pregnancy compared with WT females (Fig. 3a). Serum bile acids in neonates were also determined based on findings that maternal bile acids undergo transplacental transfer^19^,^20^. Accordingly, serum bile acids were over 421% higher in neonates from *Abcb11*^−/−^ mothers compared with those of WT, 0.059±0.011 and 0.014±0.006 mM, respectively (Fig. 3b). To address the possibility that neonatal lungs accumulate bile acids, ultra performance liquid chromatography-electrospray ionization-quadropole time-of-flight mass spectrometry (UPLC-ESI-QTOFMS) was used to measure bile acid levels in the lung. It was found that the level of tauro-muricholic acid (T-α/β-MCA) was significantly elevated in the lungs of neonates from *Abcb11*^−/−^ mothers, almost 200% higher than those from WT mothers (Fig. 3c), with no significant difference in the level of taurochenodeoxycholic acid (TCDCA; Supplementary Fig. 7).

Among the bile acid transporters, only Slco4a1 and Slco4c1 were readily detected in neonatal lungs^21^. To determine if developmental stage affected expression of *Slco4a1* and *Slco4c1*, real-time Q-PCR was performed. Lungs from foetus (E18.5) or neonate (P0) of either WT or *Abcb11*^−/−^ mothers exhibited no difference in mRNA levels of Slco4a1 and Slco4c1 (Fig. 3d). This result suggests that elevated bile acid concentration in the lungs is due to the over 400% increase in the bile acid concentration in sera of neonates from *Abcb11*^−/−^ mother and unrelated to changes in transporter expression. It was reported that bile acids can activate the MAPK pathway^22^. GSEA revealed the upregulation of the MAPK pathway in the lung of neonates from *Abcb11*^*−/−*^ mothers, which is consistent with increased pulmonary bile acid exposure (Supplementary Fig. 8a). The transcription factors Egr-1 and Ap1 are downstream of MAPK, and real-time Q-PCR demonstrated strong upregulation of Egr-1 and Ap1 target genes (Supplementary Fig. 8b,c, respectively), but only in the neonatal lungs (PN0) from *Abcb11*^*−/−*^ mothers. These findings are consistent with transplacental transfer of bile acids into the foetus (depicted in the model, Fig. 3f).

Primary bile acids are elevated in cholestasis in humans^23^,^24^. To investigate the ability of bile acids to disrupt surfactant structural order, we developed an assay where liposomes composed of a phospholipid mixture containing the major lipid constituents of lung surfactant are challenged with bile acids. These surfactant-like liposomes encapsulated an anionic fluorescent dye, 8-aminonaphthalene-1,3,6-trisulfonic acid (ANTS) and a fluorescent quencher, p-xylene-bis-pyridinium bromide (DPX) that when disrupted, released the quencher permitting ANTS to fluoresce. We interrogated these liposomes with primary and taurine conjugated bile acids found in humans and rodents (Fig. 3e). The results showed that taurine conjugated primary bile acids were more potent than unconjugated ones. Among these bile acids, TCDCA was the most effective with an EC_50_ of 75 μM. However, the unconjugated primary bile acid, chenodeoxycholic acid was also very potent (EC_50_=145 μM).

### Loss of *Nr1i2* rescued newborns

**Figure 4:**
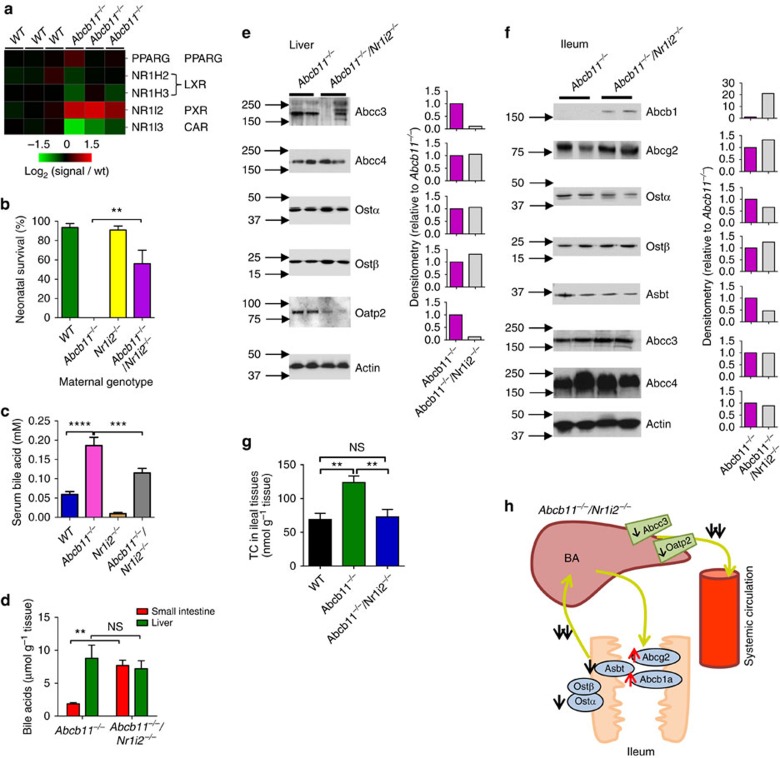
The absence of *Nr1i2* in *Abcb11*^−/−^ deficient mothers rescues neonates. (**a**) A heatmap of transcription factors, PPARγ, LXR and PXR (*Nr1i2)* in the liver revealed only PXR upregulation in *Abcb11*^−/−^ mice. (**b**) Survival rate of neonates shown with respect to their maternal genotype, WT (green bar; *n*=2), *Abcb11*^−/−^ (*n*=4), *Nr1i2*^−/−^ (yellow bar; *n*=4) and *Abcb11*^−/−^/*Nr1i2*^−/−^ (purple bar; *n*=4); *n* is the number of independent matings. All mice were congenic as defined by backcrossing at least 10 generations and SNP markers. (**c**) Serum bile acid concentrations were determined in animals of the following genotypes: WT (blue bar; *n*=15), *Abcb11*^−/−^ (purple bar; *n*=18), *Nr1i2*^−/−^ (brown bar; *n*=7), *Abcb11*^−/−^/*Nr1i2*^−/−^ (grey bar; *n*=25). (**d**) Bile pool sizes in liver and small intestine including gall bladder were determined separately in the female mice of *Abcb11*^−/−^ and *Abcb11*^−/−^/*Nr1i2*^−/−^. *n*=3. (**e**) Protein expression of hepatic transporters were measured by immunoblotting from total liver homogenates of *Abcb11*^−/−^ (purple bar) and *Abcb11*^−/−^/*Nr1i2*^−/−^ (grey bar) mice. Densitometry is on the right. (**f**) Protein expression of bile acid transporters on the apical and basolateral membranes were determined by immunoblotting in the ileal fragments of *Abcb11*^−/−^ and *Abcb11*^−/−^/*Nr1i2*^−/−^ mice. Densitometry is shown on the right. (**g**) Bile acid reabsorption in ileal fragment of small intestine was measured using a probe bile acid ^14^C-taurocholic acid. *n*=4–7. (black bar=WT, green bar=*Abcb11*^−/−^ and blue bar=*Abcb11*^−/−^/*Nr1i2*^−/−^). (**h**) A schematic model showing the changes of ileal bile acid transporters involved in enterohepatic circulation in the *Abcb11*^−/−^/*Nr1i2*^−/−^ female mice. Black single arrows indicate decreased protein expression, and red single arrows increased protein expression. Double black arrows indicate decreased bile acid transport. All experimental values are presented as mean±s.e.m. The height of the error bar=1 standard error. Statistical analysis was processed using two-tailed Student's *t*-test. **P<0.01; ***P<0.001; ****P<0.0001.

Because *Nr1i2* (PXR) affects bile acid metabolism^25^ and is the only nuclear receptor that is increased in *Abcb11*^−/−^ livers (Fig. 4a), we hypothesized that the maternal absence of both *Nr1i2* and *Abcb11*^−/−^ might affect maternal bile acid concentrations. *Abcb11*^−/−^ animals were intercrossed with congenic *Nr1i2*^−/−^ mice to produce *Abcb11*^−/−^/*Nr1i2*^−/−^ mice. The results showed that over 64% of neonates survived when their mothers lacked both *Abcb11*^*−/−*^ and *Nr1i2*^−/−^ (Fig. 4b). Notably, maternal deficiency in both *Abcb11*^*−/*−^ and *Nr1i3* (CAR) did not rescue neonatal survival as over 84% of neonates died. Associated with the increased neonatal survival was a strong reduction in serum bile acid concentration in *Abcb11*^−/−^/*Nr1i2*^−/−^ mothers compared with that in *Abcb11*^−/−^ mothers, 0.11±0.012 and 0.18±0.02 mM (*P*<0.001, Student's *t*-test), respectively (Fig. 4c). Placental bile acid transporter expression was not significant between *Abcb11*^−/−^/*Nr1i2*^−/−^ and *Abcb11*^−/−^ placentas (Supplementary Fig. 9). This dramatic increase in survival with a 39% reduction in serum bile acid was consistent with the steep dose-response curve for bile acid disruption of surfactant structure.

To investigate the mechanism of reduced serum bile acids in *Abcb11*^−/−^/*Nr1i2*^−/−^ mothers, we determined bile acid concentration in liver and small intestine tissues of *Abcb11*^*−/−*^*/Nr1i2*^*−/−*^ mothers and *Abcb11*^−/−^ mothers. Hepatic bile acid concentrations in *Abcb11*^−/−^ and *Abcb11*^−/−^/*Nr1i2*^−/−^ mothers were almost identical (Fig. 4d). Further, hepatic bile composition revealed no significant differences in T-α/β-MCA, taurocholic acid, β-MCA, TMDCA, TUDCA, TCDCA or DCA, between *Abcb11*^−/−^ and *Abcb11*^−/−^/*Nr1i2*^−/−^ mothers (Supplementary Fig. 10). An additional explanation for the reduction in serum bile acid in the *Abcb11*^−/−^/*Nr1i2*^−/−^ is the downregulation in both hepatic Abcc3 and Oatp2, both capable of exporting bile acids from the liver (Fig. 4e).

### Ileal transporter expression

The bile acid concentration in the small intestine was over 405% higher in *Abcb11*^−/−^/*Nr1i2*^−/−^ mothers (7.7±0.8 μmol g^−1^ tissue) than that in *Abcb11*^−/−^ mothers (1.9±0.2 μmol g^−1^ tissue) (*P*<0.01, Student's *t*-test; Fig. 4d). We hypothesized that this increase was, in part, due to a change in bile acid reabsorption. A mechanism accounting for the higher intestinal accumulation of bile acids in *Abcb11*^−/−^/*Nr1i2*^−/−^ might involve altered transporter expression. We determined transporter expression in the ileum of *Abcb11*^−/−^/*Nr1i2*^−/−^ and *Abcb11*^−/−^ mice (Fig. 4f). In the ileum, the primary bile acid uptake carrier, Asbt (Slc10a2) was strongly reduced by 54% in *Abcb11*^−/−^/*Nr1i2*^−/−^ compared with *Abcb11*^−/−^. Moreover, the ileal expression of the obligate heterodimeric partner of Ostβ, Ostα was reduced 35%. In combination, reduced ileal expression of luminal Asbt and basolateral Ostα likely accounts for reduced serum bile acids, especially when superimposed on the strong elevation in two apically localized ABC transporters (Abcg2 and Abcb1), capable of transporting bile acids^26^,^27^,^28^ (Fig. 4f).

### Decreased ileal bile acid transport in *Abcb11*^*−/−*^*/Nr1i2*^*−/−*^

The changes in ileal bile acid transporters support a mechanism whereby intestinal bile acid reabsorption is reduced in the *Abcb11*^−/−^/*Nr1i2*^−/−^ mothers producing lower serum bile acid concentrations. To investigate this mechanism, we used the ileal everted gut sac model^29^, assessing reabsorption of bile acids in WT, *Abcb11*^*−/−*^ and *Abcb11*^*−/−*^*/Nr1i2*^*−/−*^ females (Fig. 4g). The mucosal-to-serosal transport of taurocholate in *Abcb11*^*−/−*^*/Nr1i2*^*−/−*^ (73.0±10.8 nmol g^−1^ per 30 min) was reduced by almost 41% compared with *Abcb11*^*−/−*^ (124±9.67 nmol g^−1^ per 30 min), a level that was comparable to WT mice (69.1±9.10 nmol g^−1^ per 30 min; Fig. 4g). This indicates that bile acid reabsorption in *Abcb11*^*−/−*^*/Nr1i2*^*−/−*^ was normalized to the level found in WT females compared with *Abcb11*^*−/−*^ females.

### *Abcb11*^*−/−*^*/Nr1i2*^*−/−*^ have increased faecal bile acid excretion

The physical appearance of the faeces was no different between WT, *Abcb11*^*−/−*^ and *Abcb11*^*−/−*^*/Nr1i2*^*−/−*^. We next investigated if bile acid excretion was different among these strains. Bile acids were extracted from faecal samples using ^14^C cholic acid as internal standard. Excretion of bile acids was about twofold lower in *Abcb11*^*−/−*^ (8.60±0.62 μmol per day per 100 g BW) than WT (17.22±0.98 μmol per day per 100 g BW). Bile acid excretion was 20% higher in *Abcb11*^*−/−*^*/Nr1i2*^*−/−*^ (10.71±0.56 μmol per day per 100 g BW) mice than in *Abcb11*^*−/−*^ mice.

The mechanisms underlying neonatal respiratory distress have been largely undetermined despite an incidence of about 30% among ICP pregnancies^10^. Mechanisms accounting for cholestasis-induced neonatal respiratory distress and asphyxia are complicated and controversial^10^. One proposed mechanism posits that aspiration of bile acids during birth causes respiratory distress^30^. This is possible, but unlikely, because it does not account for the reported empirical relationship showing higher maternal bile acid concentration and longer duration of foetal exposure, which predicts neonatal pulmonary complications^10^. This relationship suggests that sustained high concentration of maternal bile acids increases foetal exposure and, consequently, pulmonary defects. This is consistent with maternal-to-foetal transplacental bile acid transfer^19^. Our finding that elevation of neonatal serum and pulmonary bile acids is positively associated with maternal serum bile acid levels supports this concept, as illustrated in Fig. 3f. The neonatal lungs, with elevated bile acids, display no evidence of overt histological damage or infiltration of pro-inflammatory cells, consistent with studies from humans^31^. But the alveoli are collapsed and remarkably similar to findings with rabbits where intratracheal instillation of bile acid produced surfactant failure^32^. We show that the protein and phospholipid composition of surfactant from affected neonates is unchanged, which agrees with the absence of change in genes regulating surfactant synthesis. The disrupted surfactant structure, evidenced by ‘tubular myelin' absence in lungs of neonates from mothers with cholestasis, is compatible with our findings that strongly elevated primary bile acid (during maternal cholestasis^1^,^2^,^3^) disrupts the structure of surfactant, producing surfactant failure. The absence of functional surfactant is consistent with our findings of pulmonary hypoxia and neonatal death. Mechanistically, our studies uncover how pulmonary bile acids induce surfactant failure and suggest that simple strategies using instilled surfactant to rescue neonates experiencing respiratory distress secondary to maternal cholestasis may not be fruitful, and suggest other approaches might be necessary. This is underscored by our findings that very small changes in bile acid concentration readily disrupt surfactant lipid structure.

Disruption of *Nr1i2 (PXR)* in the *Abcb11*^−/−^ mothers raises newborn survival from 0% to over 60%. This is due to decreased systemic bile acid concentrations in the *Abcb11*^−/−^/*Nr1i2*^−/−^ mothers compared with *Abcb11*^−/−^ mothers. Although bile acids could be excreted from the systemic circulation to the urine via renal transporters system, renal bile acid excretion is not substantially different between *Abcb11*^−/−^ and *Abcb11*^−/−^/*Nr1i2*^−/−^ mothers (Supplementary Fig. 11). This is because bile acid concentrations in the urine and expression of key bile acid transporters were not significantly different between *Abcb11*^−/−^/*Nr1i2*^−/−^ and *Abcb11*^−/−^ animals (Supplementary Fig. 11). The importance of the *Nr1i2* (*PXR*) as a modulator of ICP in humans is supported by recent genomic studies showing an increased frequency of both a PXR variant^33^ as well as altered PXR promoter methylation in ICP patients^34^. Combined with our current findings, the role of *Nr1i2* (*PXR*) in modulating serum bile acids in ICP may have been underestimated.

In total, these studies reveal how, in maternal cholestasis, bile acids impact neonatal survival by directly interfering with surfactant function. In light of this strong effect, these studies have important implications, not just for genetic factors that affect maternal bile acid levels, but also environmental factors such as drugs that produce cholestasis secondary to inhibition of bile acid transporters (for example, ABCB11). Mechanistically, maternal absence of the nuclear receptor *Nr1i2* reduced maternal serum bile acid concentrations by reducing intestinal reuptake of bile acids. Beside the Nr1i2 pathway, another potential therapeutic pathway that could be exploited to improve neonatal health is the ileal bile acid uptake pathway. In this respect, however, we propose that reducing intestinal bile acid reuptake, perhaps by inhibition of bile acid uptake by either Asbt or Ostβ and Ostα might be effective. The current studies have provided a model to test this approach and provide an important conceptual framework enabling future strategies to enhance neonatal survival.

## Methods

### Animals

*Abcb11*^*−/−*^ mice on C57BL/6 genetic background were purchased from Jackson Laboratory. *Nr1i2*^*−/−*^ mice were backcrossed 10 generations into a C57BL/6 genetic background and intercrossed with *Abcb11*^*−/−*^ mice to produce *Abcb11*^−/−^/*Nr1i2*^−/−^ mice. Mice were maintained in a temperature and humidity controlled room with free access to water and food with 12/12 light and dark cycles. All procedures were reviewed and approved by the Institutional Animal Care and Use Committee of St Jude Children's Research Hospital.

The VisualSonics VEVO-2100 High-Resolution Imaging System was used to monitor gestational stage. The number of dead and live neonatal pups was recorded at the day of birth for determination of litter size and neonatal mortality. Only live pups were weighed within 12 h after birth.

Small intestine tissues (described above) were equally cut into three equal-sized fragments with the last fragment used representing the ileum, based on Asbt expression^29^, that was snap frozen in liquid nitrogen and stored at −80 °C for western blot and real-time Q-PCR. Placenta tissues were collected at E17.5 after C-section.

### Measurement of bile acid pool size

Liver and small intestine (including contents and gall bladder) were removed from mice and weighed separately. Bile acid pool size was measured as described by Wooton-Kee *et al*.^35^ After the addition of glycocholic acid (6 μmol per 50 ml ethanol), the tissues were minced and then homogenized in this ethanol solution (12.5 ml g^−1^ tissue) followed by an incubation in a shaking water bath (60 °C). The particulate matter was removed by filtration and the remaining extract was dried under a stream of N_2_ and then resuspended in methanol. Any remaining particulate was removed by first, centrifugation at 14,000*g* for 10 min, followed by filtration through polyvinylidene difluoride membrane (0.45 μM). The filtrate was dried and then dissolved in 1 ml of 75% methanol. The final bile acid extract was analysed by high-performance liquid chromatography.

### Serum bile acid measurement

Blood was collected from mothers retro-orbitally 1 week before mating (P0) and day 15 during pregnancy (P15) and from neonatal pups after decapitation within 12 h after birth (PN0). Total bile acids in serum were measured using an enzymatic assay^36^.

### Measurement of bile acid composition in lung

Lung tissues were dissected after decapitation within 12 h after birth, snap frozen in liquid nitrogen and stored at −80 °C. To determine the concentration of T-α/β-MCA in neonatal lung tissues, lung samples (about 50 mg) were minced in 10-fold 50% aqueous acetonitrile and shaken for 15 min at room temperature. The homogenate was centrifuged at 14,000*g* for 20 min, and a 5-μl aliquot of supernatant was injected into an UPLC-ESI-QTOFMS system (Waters Corporation). The concentration of T-α/β-MCA in the homogenate was determined against calibration curves constructed with authentic standards (0–50 μM). The correlation coefficient was >0.99. Chlorpropamide, at a final concentration of 5 μM, was used as the internal standard.

### Measurement of bile acid composition in liver

Liver samples (20 mg) were homogenized with 200 μl 100% acetonitrile containing 2 μM taurocholic acid-d5 as internal standard. The homogenate was incubated at room temperature for 10 min following by centrifugation at 13,000 r.p.m. × 10 min. The supernatant was diluted 100-fold by acetonitrile/water/formic acid (20/80/0.1). For bile acids quantitation, a 5-μl aliquot of dilution was introduced into Waters Acquity H-class UPLC system using a Waters Acquity BEH C18 column (2.1 × 100 mm) coupled to a Waters Xevo G2 QTOF mass spectrometer. UPLC: A-0.1% formic acid in water, B-0.1% formic acid in acetonitrile. Gradient: initial 80% A for 4 min, to 60% A at 15 min, to 40% A at 20 min, to 10% A at 21 min. Flush for 1 min, then equilibrate at initial conditions for 4 min. Flow rate 0.4 ml min^−1^. Column temperature was maintained at 45 °C. Waters Xevo G2 QTOF was operated in negative mode, scanning 50–850 AMU, at a rate of 0.3 scans per sec. The following instrument conditions were used: capillary 1.5 kV, source temp 150 °C, sampling cove 30 V, desolvation gas flow 850 l h^−1^ at 500 °C. Bile acids standards were prepared from 0.003 to 10 μM. Standard curves with correlation coefficients >0.99 were used to measure the concentration of bile acids in tissue samples.

### Immunoblot analysis

Tissue homogenates were used to determine protein expression by immunoblot. The blots were blocked and incubated with the primary antibodies. The Asbt, Ostα and Ostβ antibodies were kindly provided by Dr Paul Dawson (Emory University). The Aqp5 antibody was obtained from Alomone Labs (Jerusalem, Israel), and the Meca32 and Abca3 antibodies from Santa Cruz Biotechnology (Santa Cruiz, CA). The Mrp2 antibody M2III-5 was purchased from Enzo life Science (Plymouth Metting, PA), while the Pro-Sftpc, Sftpc and Sftpb were from Seven Hills Bioreagents (Cincinnati, OH). Appropriate horseradish peroxidase–linked secondary antibodies (Amersham Bioscience) were used according to their individual primary antibodies. The immunoblots were developed using the Amersham enhanced chemiluminescence detection system. The uncropped scans of the immunoblots are presented in the Supplementary Fig. 12.

### Immunohistochemistry

On neonatal pups decapitation, whole lungs were dissected and fixed in 10% formalin. Routine H&E staining was performed and other immunohistochemistry with antibodies to Sftpc, (Santa Cruz Biotechnology, Santa Cruz, CA), Caspase 3 and Ki67.

### Electronic microscopy

Lungs of neonatal pups were perfused *in situ* with fixative solution (2% paraformaldehyde, 2.5% gluteraldehyde in CaCO_4_ without sucrose), and sectioned for electronic microscopy.

### Microarray analysis and real-time Q-PCR

RNA was isolated using RNeasy Kit (Qiagen) from the lungs of neonatal pups, according to the manufacturer's protocol. The quality and integrity of the RNA was confirmed by analysis on an Agilent 2100 Bioanalyzer. Biotin-labelled targets, prepared from 100 ng total RNA using the Affymetrix 3'IVT express protocol, were hybridized to mouse HT MG430 PM plate arrays and then processed automatically using the Affymetrix GeneTitan system. Signals from scanned arrays were summarized using the robust multiarray averaging method^37^,^38^. GSEA^37^,^38^ was performed using curated canonical pathways obtained from MolSigDB (Broad Institute), Ingenuity Systems (www.ingenuity.com) and GeneGO (Thomas Reuters, Inc). The same RNA was used in Q-PCR analysis for confirmation.

### Membrane destabilization of surfactant-like liposomes

Liposomes (4 mg) were made using 1-palmitoyl-2-oleoyl-*sn*-glycero-3-phosphocholine, 1,2-dipalmitoyl-*sn*-glycero-3-phosphocholine and 1,2-dioleoyl-sn-glycero-3-phospho-(1'-rac-glycerol) 5:4:1 (w/w/w). The lipids were combined in a glass tube and blown down to dryness under N2. ANTS (12.5 mM) and DPX (45 mM) were dissolved in 10 mM Tris pH 7.4, 100 mM KCl solution. ANTS (500 μl) and DPX (500 μl) were added to the dry lipids, the mixture vortexed and rehydrated at 37 °C for 1 h. After 5 min in a sonication bath, the liposomes were passed 25 times through a mini-extruder containing a 400-nm polycarbonate filter. Unincorporated ANTS/DPX was removed using a Zeba Spin Desalting column (7 K Molecular weight cut-off, 5 ml; Thermo Scientific). Bile salts stocks and dilutions were made with 10 mM Tris pH 7.4, 100 mM KCl. Assays were performed in a 96-well white OptiPlate (PerkinElmer) and contained 5 μl of the ANTS/PDX liposomes mixed with 200 μl of bile salt of different concentrations. Reactions were incubated at room temperature for 1 h in the dark. The plate was read on Fusion reader (PerkinElmer) using the following settings: emission filter, 355 nm; excitation filter, 530 nm; 5 s read per well; and PMT voltage set to high.

### Measurement of bile acid faecal excretion

Mice were acclimated to metabolic cages for 3 days before faecal sample collection, which occurred for three consecutive 24 h periods. Bile acids were extracted from faecal samples as described^39^ and total bile acids measured by an enzymatic assay^29^. Briefly, pooled faecal samples were collected over a 3 day interval dried in 80 °C oven overnight, and then ground to powder with a mortar and pestle. Duplicate 1.0 g aliquots of faecal powder were mixed in a ethanol solution containing sodium borohydride (2 mg ml^−1^). As an internal control for bile acid extraction (24-^14^C) cholic acid (0.04 μCi) was added. The samples were then mixed with HCl (2 N) followed by NaOH (10 N). The samples were then refluxed in an oil bath at 120–130 °C overnight. The samples were cooled, filtered and dried under a N_2_ stream. After resuspension in H_2_O, an aliquot was applied to a C18 column, washed and the bile acids eluted with 20% methanol. This extract was dried under N_2_, resuspended in methanol, and any remaining precipitate removed by centrifugation. An aliquot of the extract was sampled to determine the radioactivity as a means of assessing bile acid recovery. Bile acids were measured by an enzymatic assay and the daily rate of faecal bile acid excretion was calculated after normalization by BW.

### Everted gut sac transport measurement

The mucosal-to-serosal transport of taurocholate was measured as described previously^40^. After euthanization, a 7-cm segment of small intestine ileum was isolated after measuring from the beginning of the large bowel. The everted gut sac was prepared from this segment. The everted gut sac was filled with Kreb's Ringer Buffer (KRB, pH 7.4) and taurocholate transport determined (the mucosal-to-serosal direction) after immersing the everted gut sac into the mucosal fluid containing KRB supplemented with 25 μM taurocholate and a tracer ^3^H-taurocholate as well as ^14^C-inulin to normalize for paracellular leakage. The KRB bathing solution was continuously gassed with 95% O_2_/5% CO2. Radioactivity of ^3^H-taurocholate in the serosal fluid was measured after a 30 min interval.

### Statistics

All experimental values are presented as mean±s.e.m. Statistical analysis was processed using two-tailed Student's *t*-test. *P<0.05; **P<0.01; ***P<0.001; ****P<0.0001.

## Additional information

**How to cite this article:** Zhang, Y. *et al*. Maternal bile acid transporter deficiency promotes neonatal demise. *Nat. Commun.* 6:8186 doi: 10.1038/ncomms9186 (2015).

## Supplementary Material

Supplementary Information Supplementary Figures 1-12
