# Supplementary material for: Maternal bile acid transporter deficiency promotes neonatal demise
Source: Nat Commun. 2015 Sep 29;6:8186. doi: 10.1038/ncomms9186 (PMC4598356; doi:10.1038/ncomms9186)
Supplement: Supplementary Information — Supplementary Figures 1-12 [file ncomms9186-s1.pdf]

Supplementary Figure 1

WT♀ x *Abcb11*$^{-/-}$♂ **—** ***Abcb11*$^{-/-}$♀ x WT♂**

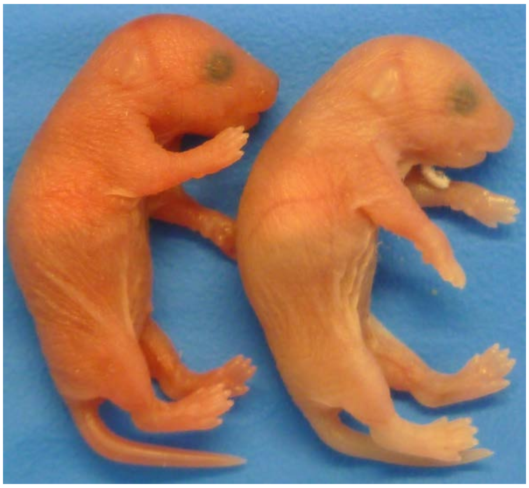

Supplementary Data Figure 1. Neonates from *Abcb11*$^{-/-}$ mothers appeared pale. A representative image of neonatal coloration from interbreeding as follows: WT♀ x *Abcb11*$^{-/-}$♂ and *Abcb11*$^{-/-}$♀ x WT♂. n>10.

Supplementary Figure 2

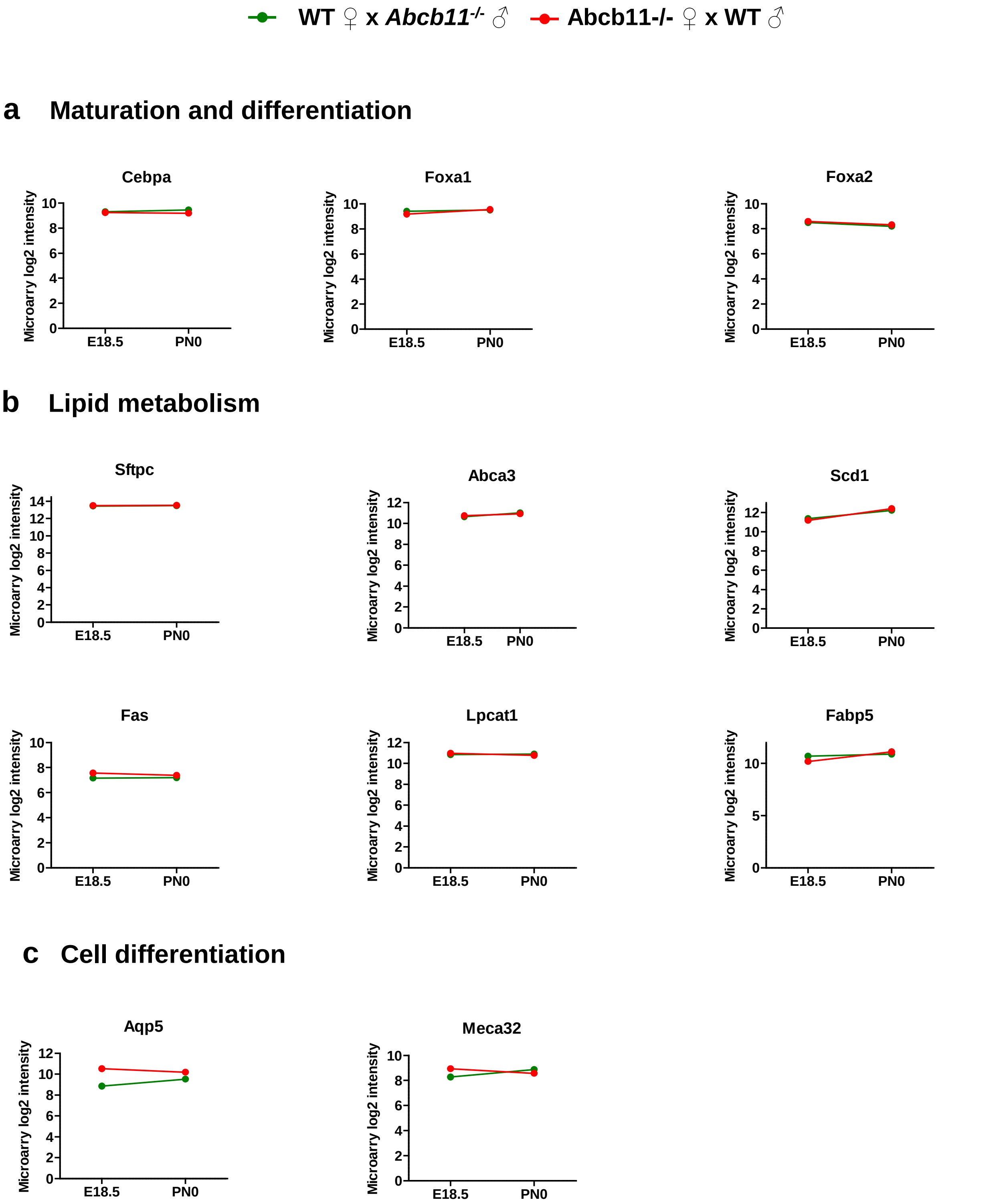

Supplementary Data Figure 2. No difference observed in lung development. Gene expression analysis was performed in lung tissues collected from embryos (E18.5, n=3) and birth day neonates (PN0, n=3) from WT and *Abcb11*$^{-/-}$ mothers. Real time Q-PCR analysis was performed in triplicate. Expression of genes representing lung maturation and differentiation (**a**), lipid metabolism (**b**), and cell differentiation (**c**). The green symbols indicate the mother's genotype was WT and the red indicate the mother was *Abcb11*$^{-/-}$.

Supplementary Figure 3

a

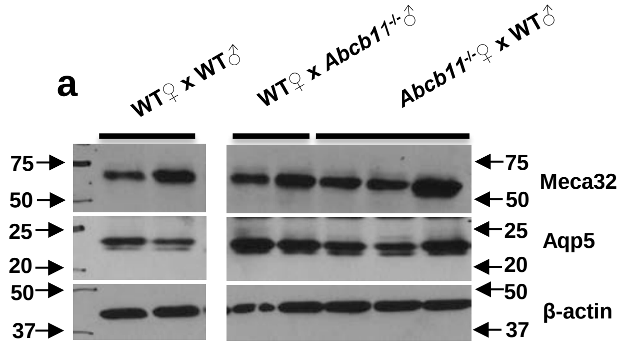

b

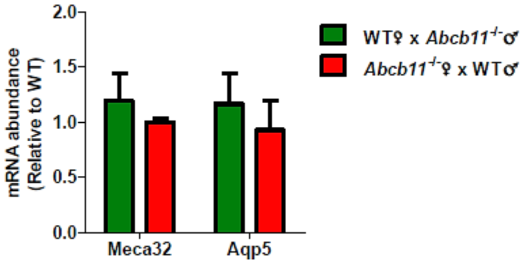

Supplementary Data Figure 3. Neonatal lungs showed no difference in cell markers. Protein and mRNA expression of markers for cell types in the neonatal lung from WT and *Abcb11*$^{-/-}$ mothers were evaluated by western blot (**a**) and real-time Q-PCR (**b**, n=3 neonates/genotype per mother). Meca32, vascular cells; Aqp5, pneumocyte type I. Western blot images were representative of at least three independent replicates. Real time Q-PCR analysis was performed in triplicate for each sample. The green bars indicate the mother's genotype was WT and the red indicate the mother was *Abcb11*$^{-/-}$. Statistical analysis was processed using two-tailed Student's t test. Data are presented as mean ± standard error of the mean (SEM).

Supplementary Figure 4

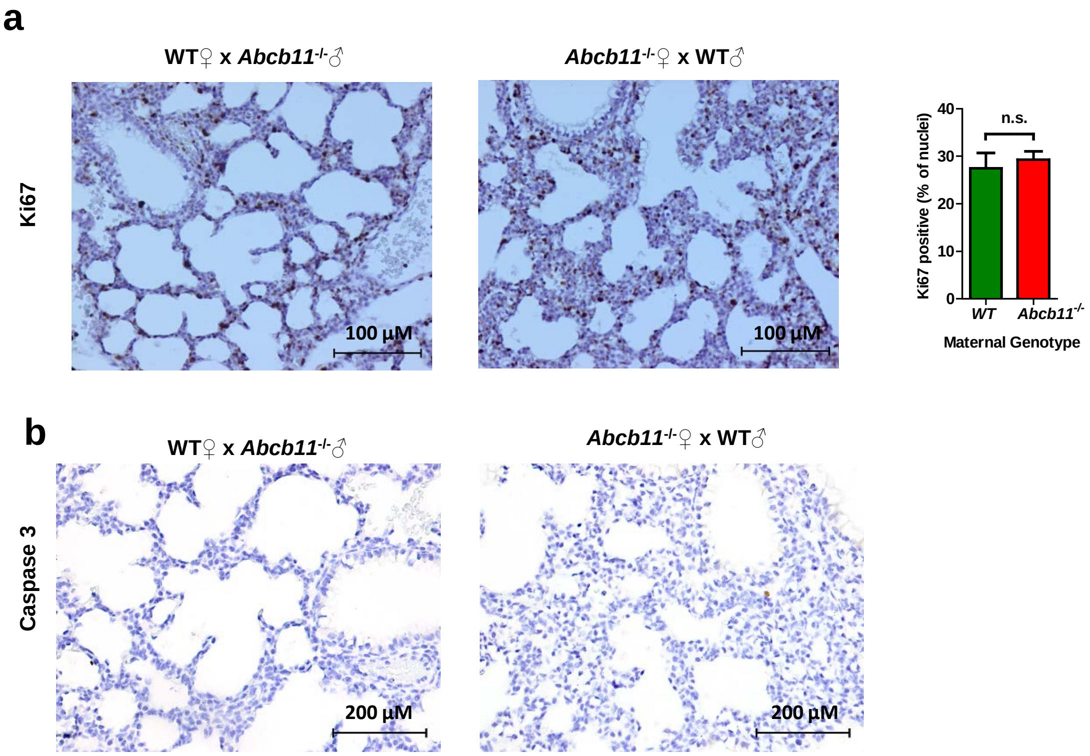

Supplementary Data Figure 4. No changes in proliferation or apoptosis of pulmonary cells in neonates from *Abcb11*$^{-/-}$ mothers. a. Proliferation (Ki67) determined in neonatal lungs by immunohistochemistry**.** Ki67 positive cells were counted using Aperio and normalized to the number of nuclei. **b**. Apoptosis (Caspase 3) determined in neonatal lungs by immunohistochemistry**.** Representative lung pictures from neonates (n>3) from WT and *Abcb11*$^{-/-}$ mothers were shown. Data are presented as mean ± standard error of the mean (SEM). Statistical analysis was processed using two-tailed Student's t test. n.s., non-statistical significance.

Supplementary Figure 5

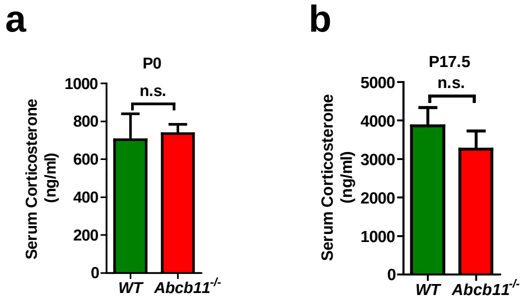

Supplementary Data Figure 5. Serum corticosterone levels in *Abcb11*$^{-/-}$ mothers were not different from those in WT mothers. Sera were collected from virgins (P0, **a**.) and gestational females (P17.5, **b.**) and corticosterone was determined by EIA kit. n=5. Data are presented as mean ± standard error of the mean (SEM). Statistical analysis was processed using two-tailed Student's t test. n.s., non-statistical significance. P0, before pregnancy; P17.5, day 17.5 of pregnancy.

Supplementary Figure 6

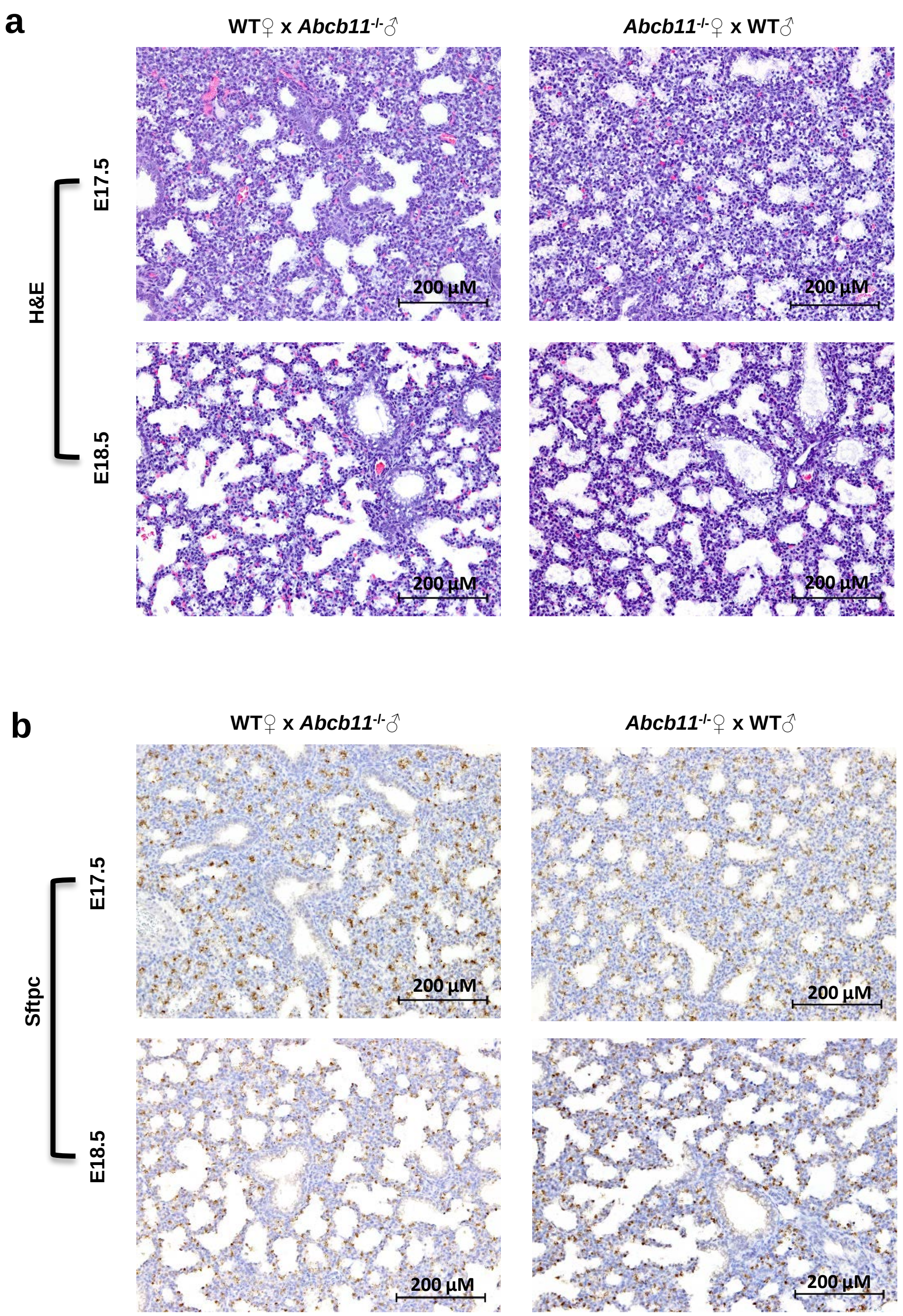

Supplementary Data Figure 6. No changes in morphology or development of embryonic lungs. Lung tissues were collected from embryos at E17.5 and E18.5 from WT and *Abcb11*$^{-/-}$ mothers for immunohistological analysis. **a.** H&E; **b.** Sftpc staining. Representative pictures were shown. The images in each group were representative of at least 3 embryos analyzed.

Supplementary Figure 7

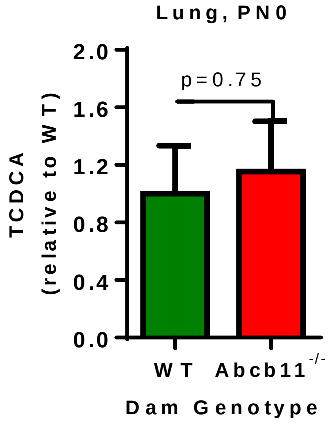

Supplementary Data Figure 7. Bile acid levels in the neonatal lungs of WT, *Abcb11*$^{-/-}$ and *Abcb11*$^{-/-}$/Nr1i2$^{-/-}$ mothers. TCDCA levels were determined in the lung tissues of neonatal offspring of WT, *Abcb11*$^{-/-}$ and *Abcb11*$^{-/-}$/Nr1i2$^{-/-}$ mothers. n=5. Data are presented as mean ± standard error of the mean (SEM). Statistical analysis was processed using two-tailed Student's t test. n.s., non-statistical significance. PN0, is the day of birth.

Supplementary Figure 8

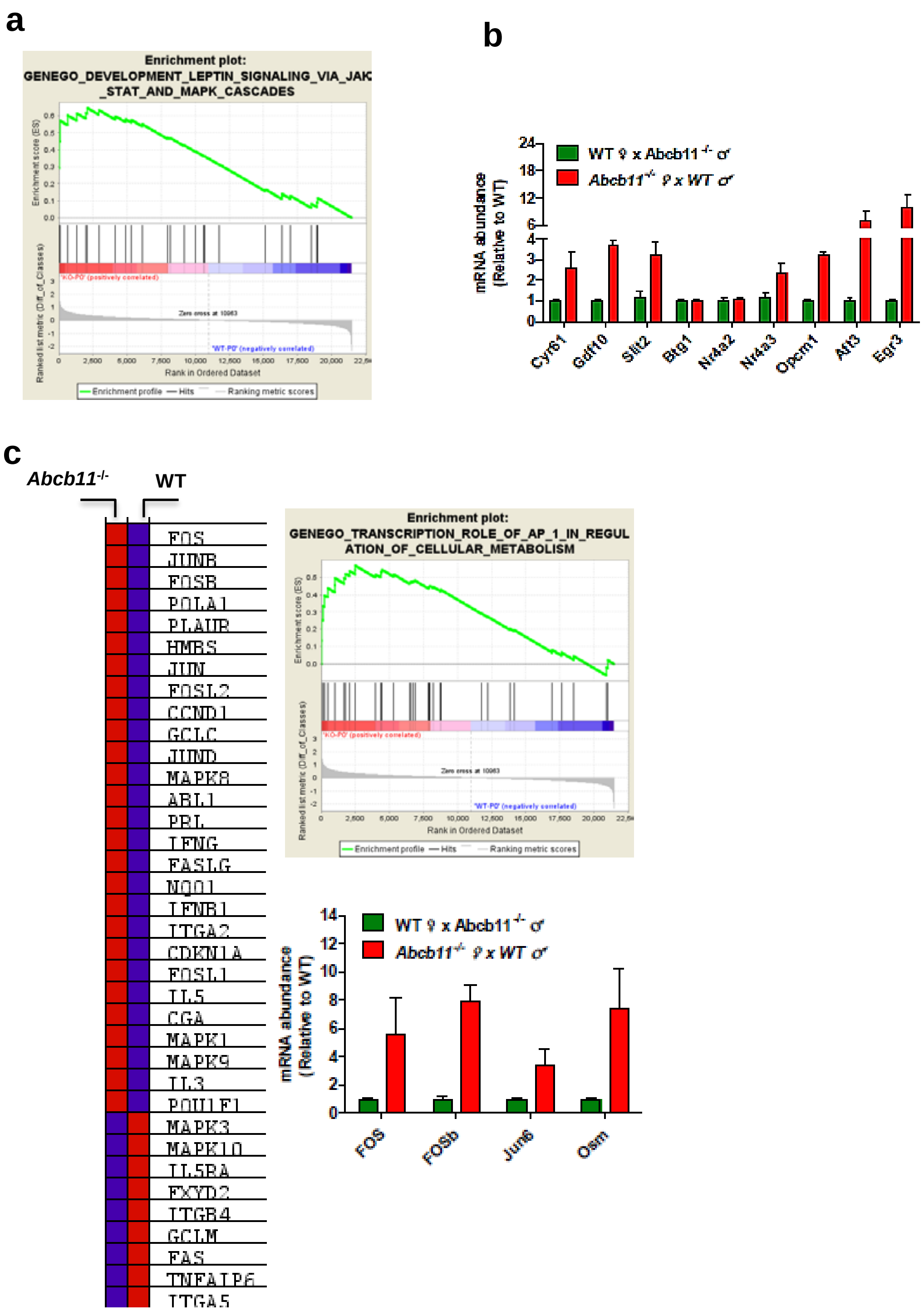

Supplementary Data Figure 8. Stress responsive gene expression was upregulated in the neonatal lungs. a. GSEA of the gene expression in the lung tissues showed activation of MAPK pathway in heterozygote neonates from *Abcb11*$^{-/-}$ mothers. **b.** The expression of Egr1 target genes determined by real time Q-PCR in the lung tissues neonates (PN0) from WT♀ x *Abcb11*$^{-/-}$♂ and *Abcb11*$^{-/-}$♀ x WT♂. n=4. **c.** Ap1 pathway was activated in the lung tissues in heterozygous neonates from *Abcb11*$^{-/-}$ mothers shown by GSEA. Real-time Q-PCR determined the expression of Ap1 genes in the lung tissues of heterozygous neonates from WT♀ x *Abcb11*$^{-/-}$♂ and *Abcb11*$^{-/-}$♀. n=4. The green bars indicate the mother's genotype was WT and the red indicate the mother was *Abcb11*$^{-/-}$.

Supplementary Figure 9

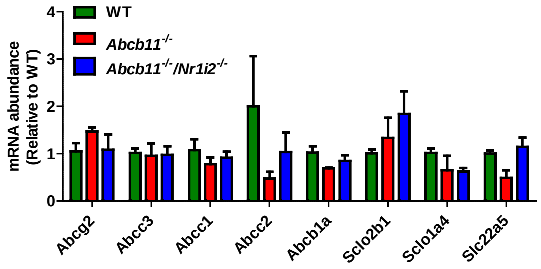

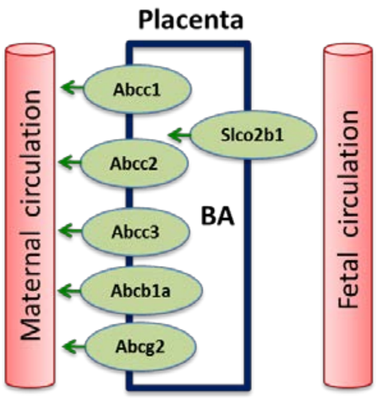

Supplementary Data Figure 9. No difference in bile acid transporters in placenta tissues. Expression of mRNA of bile acid transporters in placenta tissues from WT, *Abcb11*$^{-/-}$ and *Abcb11*$^{-/-}$/Nr1i2$^{-/-}$ mothers were evaluated by real-time Q-PCR. n=4. BA, bile acid.

Supplementary Figure 10

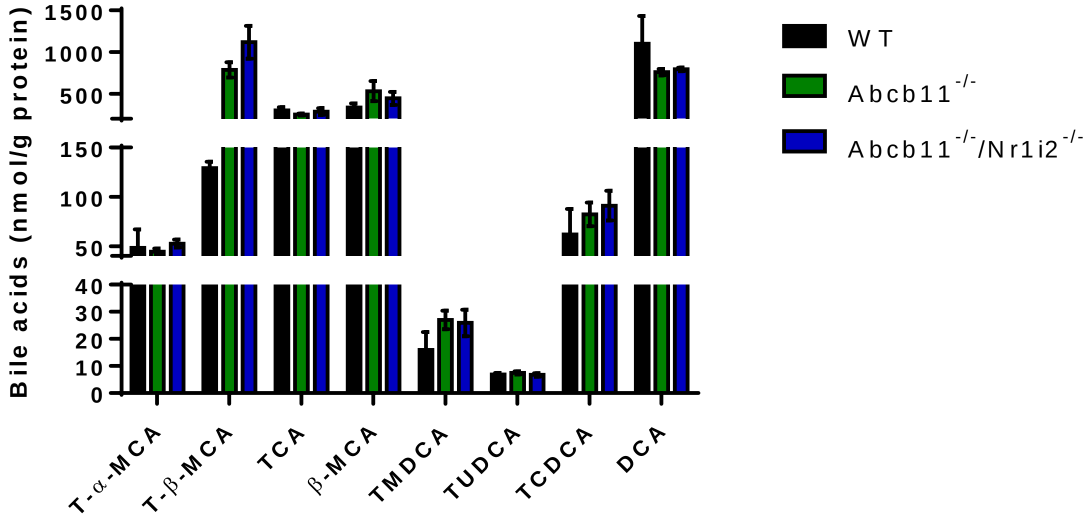

Supplementary Data Figure 10. Hepatic bile composition in WT, *Abcb11*$^{-/-}$ and *Abcb11*$^{-/-}$/Nr1i2$^{-/-}$ females. The levels of bile acids in the livers of WT, *Abcb11*$^{-/-}$ and *Abcb11*$^{-/-}$/Nr1i2$^{-/-}$ females were measured as described in the "Methods". n=5.

Supplementary Figure 11

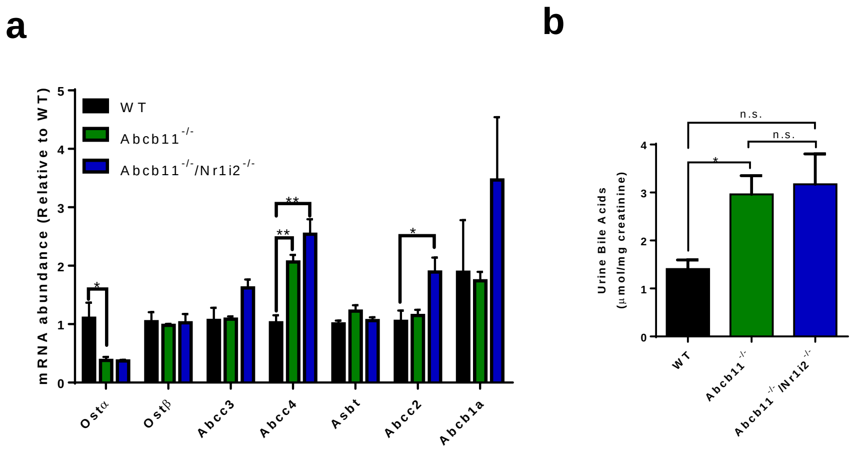

Supplementary Data Figure 11. Renal transporter expression and urinary bile acid excretion in WT, *Abcb11*$^{-/-}$ and *Abcb11*$^{-/-}$/Nr1i2$^{-/-}$ females. Expression of bile acid transporters in kidney tissue from WT, *Abcb11*$^{-/-}$ and *Abcb11*$^{-/-}$/Nr1i2$^{-/-}$ mice were evaluated by real-time Q-PCR. n=4-5. The amount of bile acid in the urine WT, *Abcb11*$^{-/-}$ and *Abcb11*$^{-/-}$/Nr1i2$^{-/-}$ females was determined as described in the "Methods" n=5-6. Statistical analysis was processed using two-tailed Student's t test. n.s., non-statistical significance. . *, p<0.05; **, p<0.01; ***, p<0.001; ****, p<0.0001.

Supplementary Figure 12

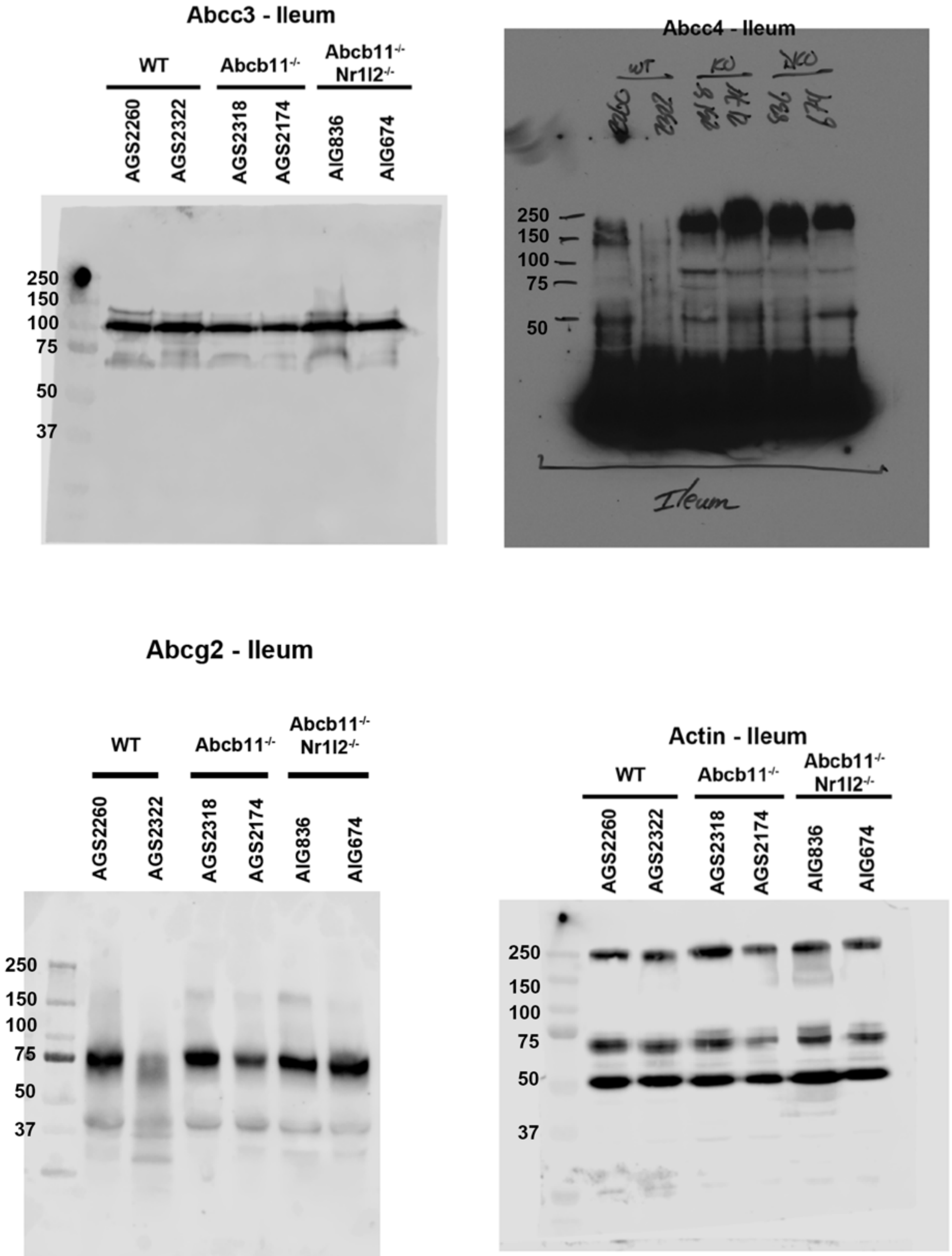

Supplementary Data Figure 12. Uncropped immunoblot images are shown (including molecular weight markers, except in reprobed blots)

Asbt - Ileum

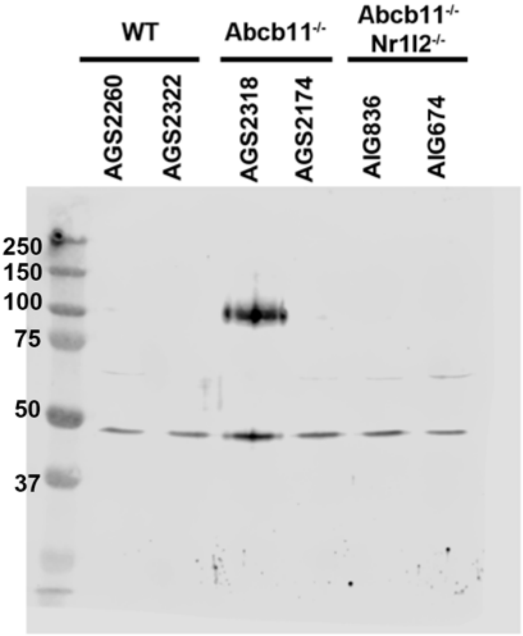

Ostα - Ileum

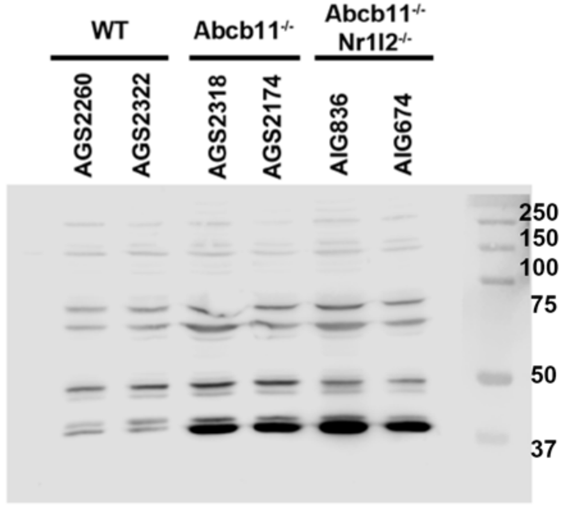

Ostβ - Ileum

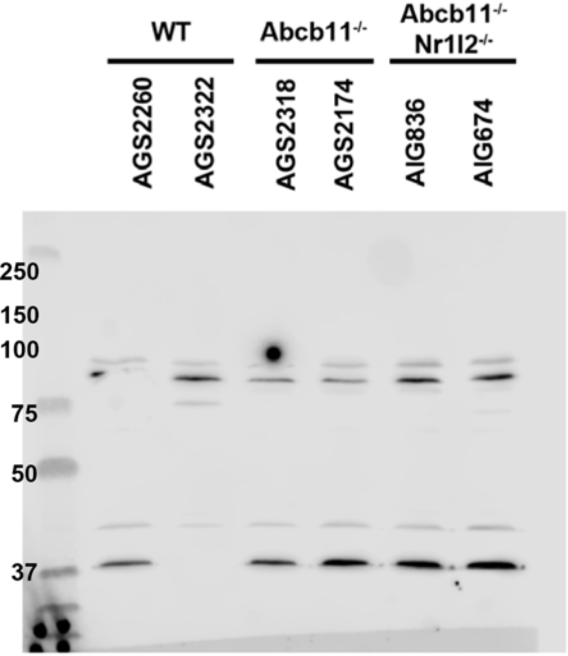

Abcb1 - Ileum

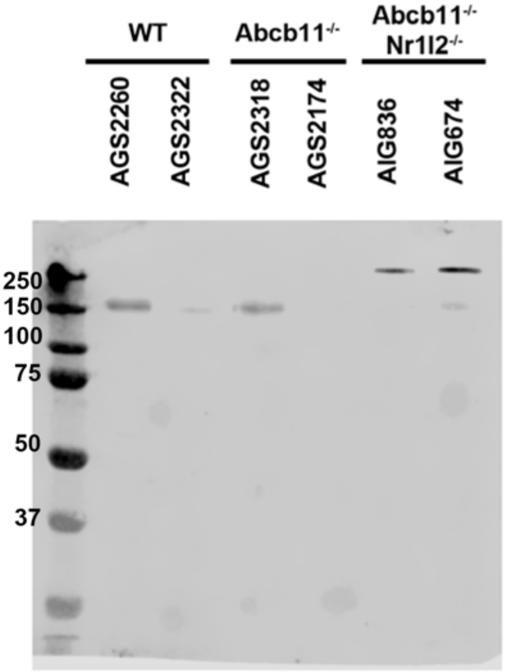

Abcb1 - Liver

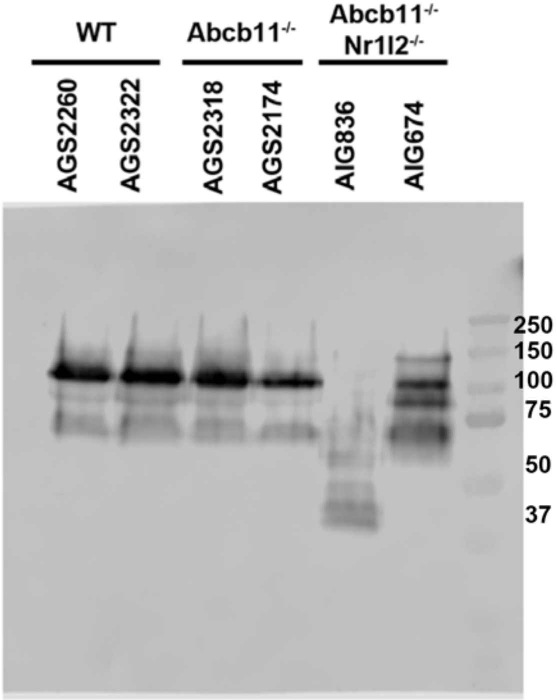

Actin - Liver

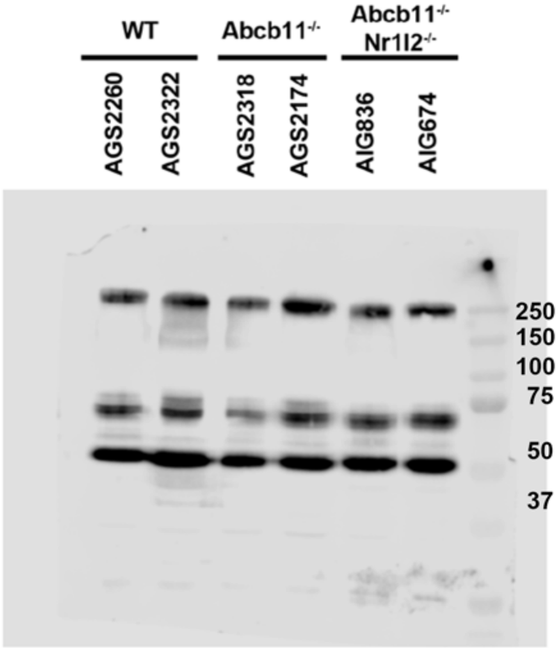

Oatp1 - Liver

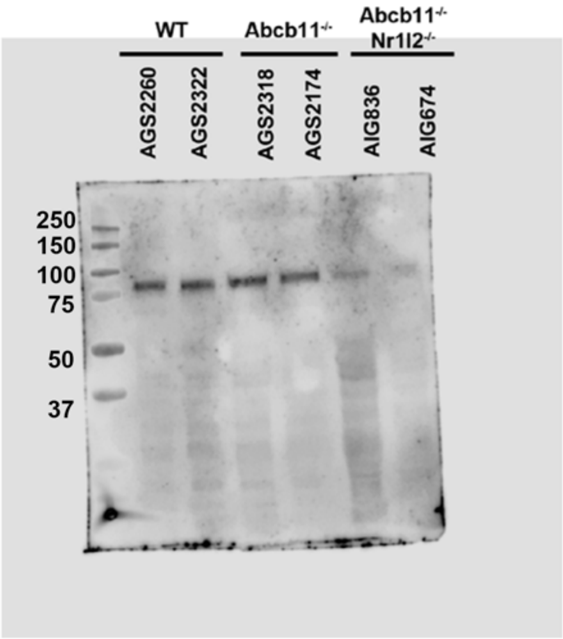

Ostα - Liver

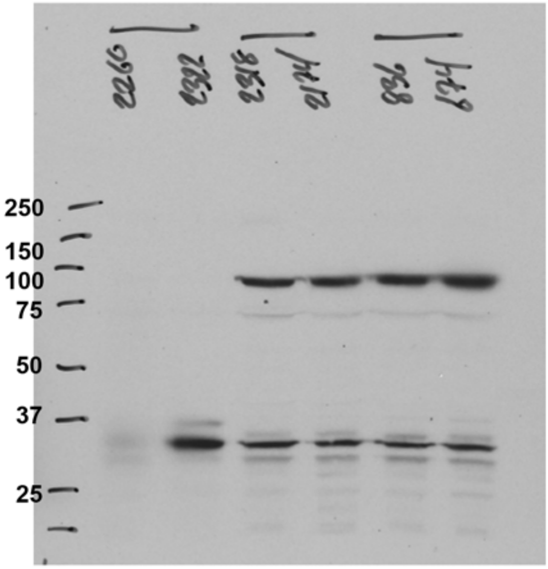

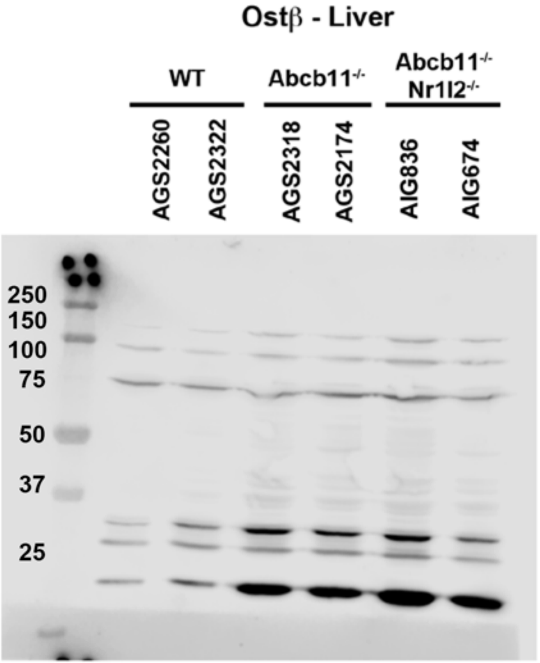

Ostβ - Liver
WT
Abcb11-/-
Abcb11-/-
Nr1I2-/-
AGS2260
AGS2322
AGS2318
AGS2174
AIG836
AIG674
250
150
100
75
50
37
25

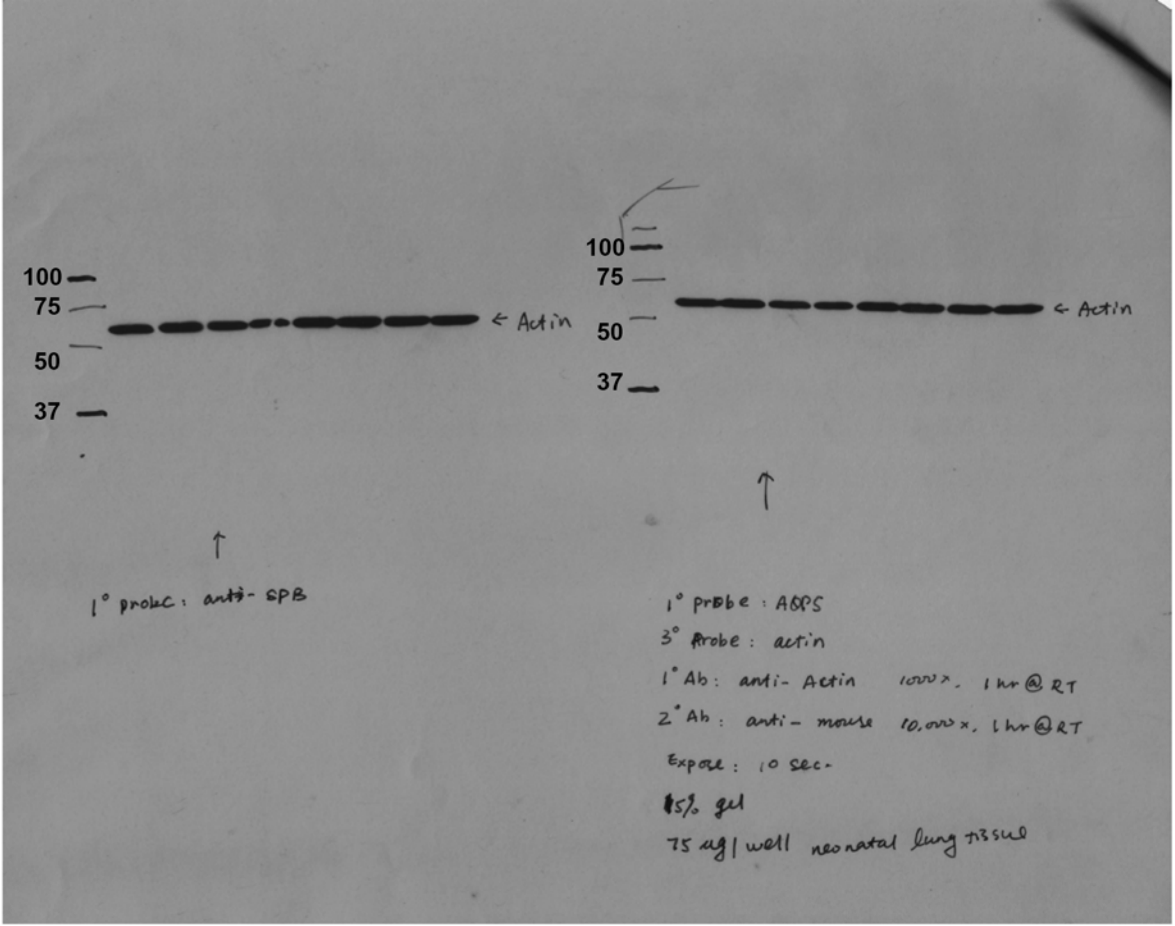
100
75
50
37
← Actin
100
75
50
37
← Actin
1° probe: anti-SPB
1° probe: AQP5
3° probe: actin
1° Ab: anti-Actin 1000x, 1hr@RT
2° Ab: anti-mouse 10,000x, 1hr@RT
Expose: 10 sec.
15% gel
75 ug/well neonatal lung tissue

ACTIN – Lung

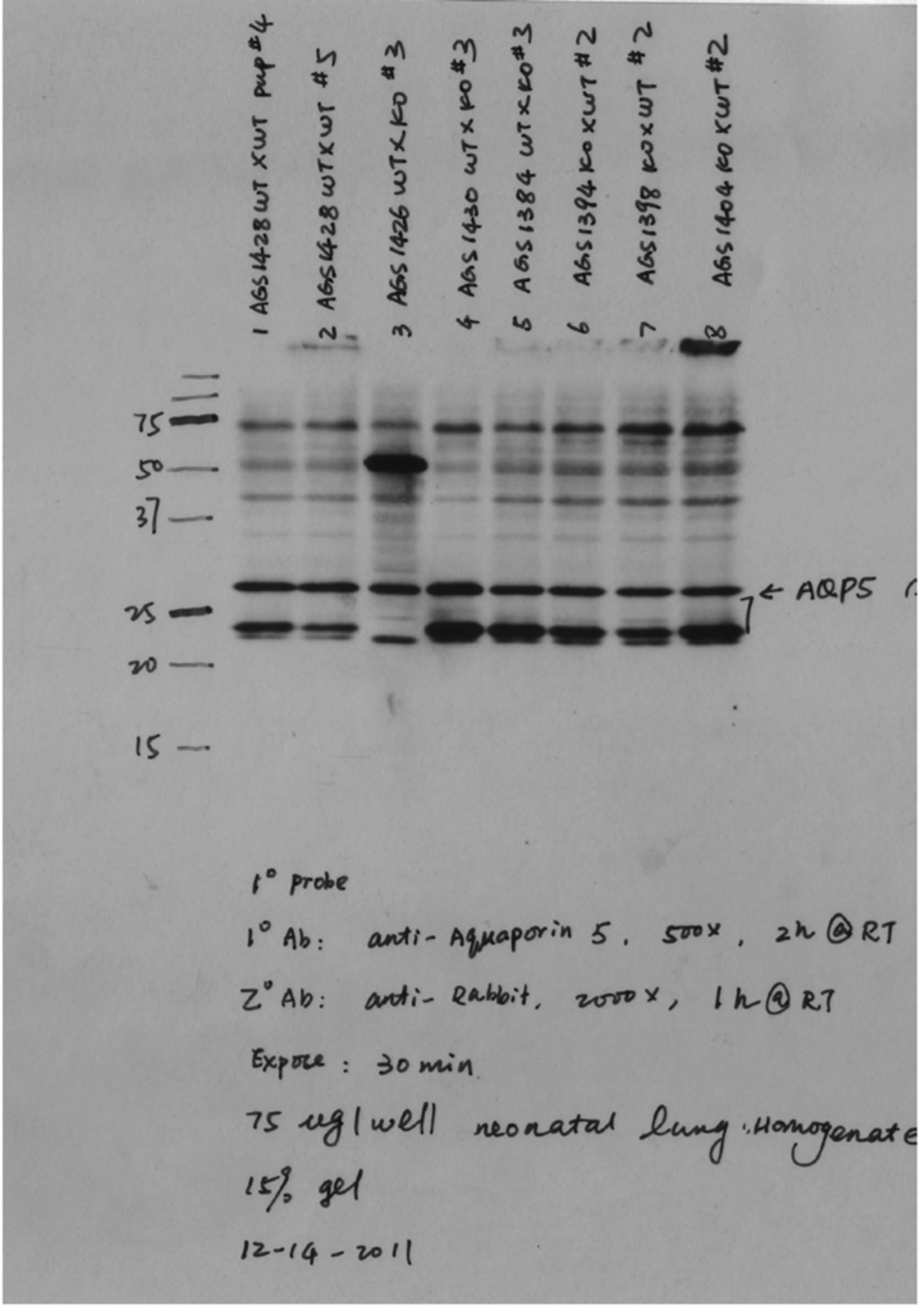
1 AGS1428 WT X WT pup #4
2 AGS1428 WT X WT #5
3 AGS1426 WT X KO #3
4 AGS1430 WT X KO #3
5 AGS1384 WT X KO #3
6 AGS1394 KO X WT #2
7 AGS1398 KO X WT #2
8 AGS1404 KO X WT #2
75
50
37
25
20
15
← AQP5
1° probe
1° Ab: anti-Aquaporin 5, 500x, 2h @ RT
2° Ab: anti-Rabbit, 2000x, 1h @ RT
Expose: 30 min
75 ug/well neonatal lung Homogenate
15% gel
12-14-2011

AQP5 – Lung

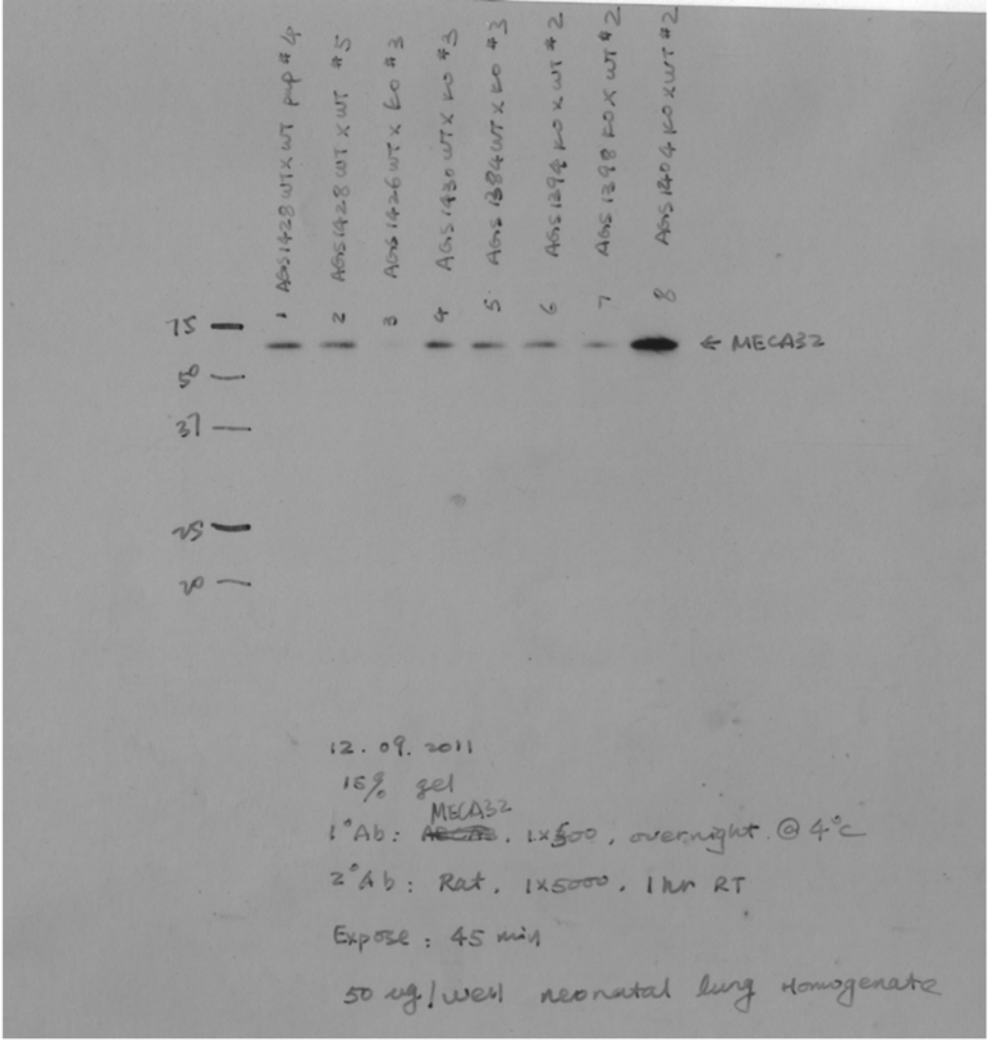
AGS1428 WT X WT pup #4
AGS1428 WT X WT #5
AGS1426 WT X KO #3
AGS1430 WT X KO #3
AGS1384 WT X KO #3
AGS1394 KO X WT #2
AGS1398 KO X WT #2
AGS1404 KO X WT #2
1
2
3
4
5
6
7
8
75
50
37
25
20
← MECA32
12.09.2011
15% gel
1°Ab: MECA32, 1X500, overnight @ 4°C
2°Ab: Rat, 1X5000, 1hr RT
Expose: 45 min
50 ug/well neonatal lung Homogenate

MECA32 – Lung

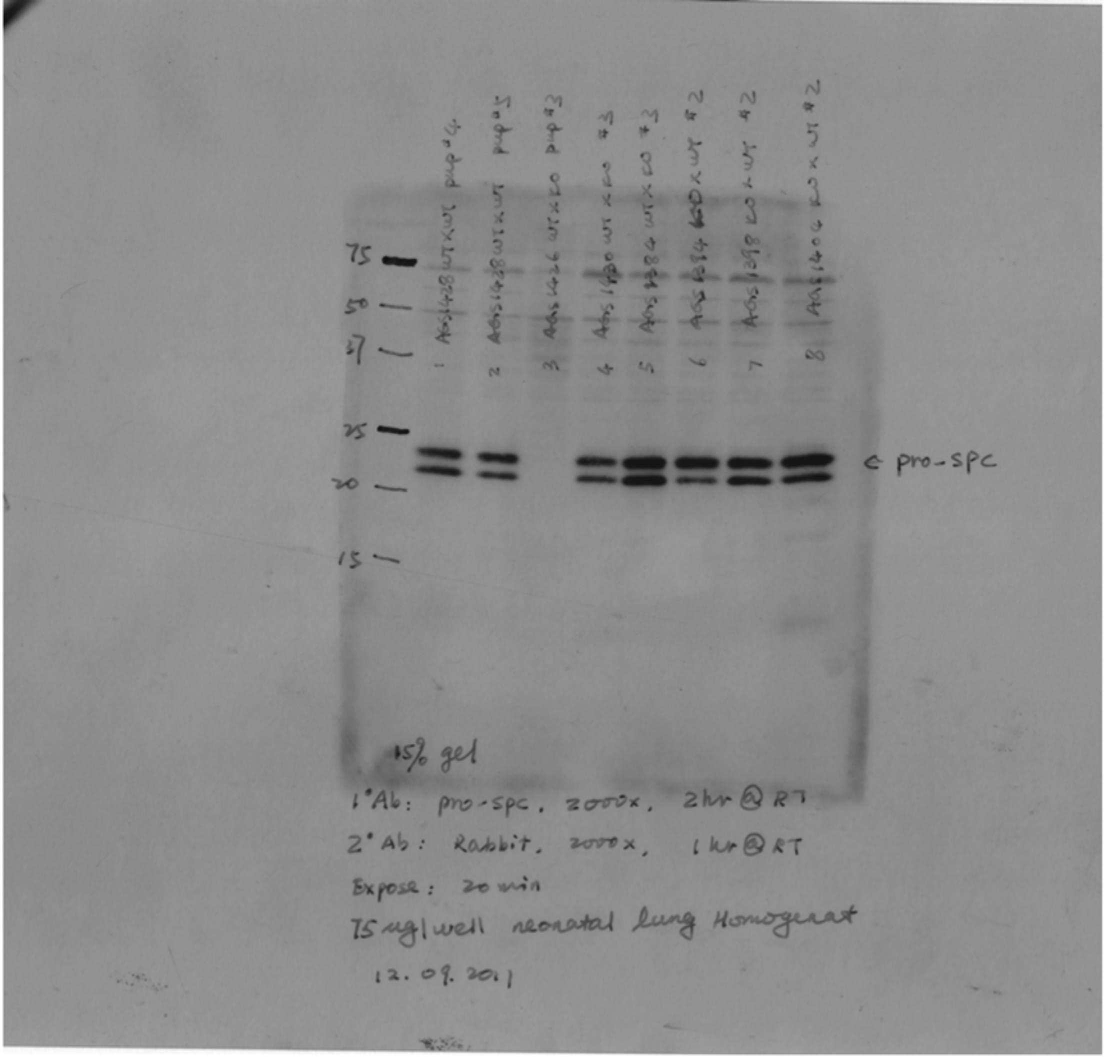

Pro-SftPC – Lung

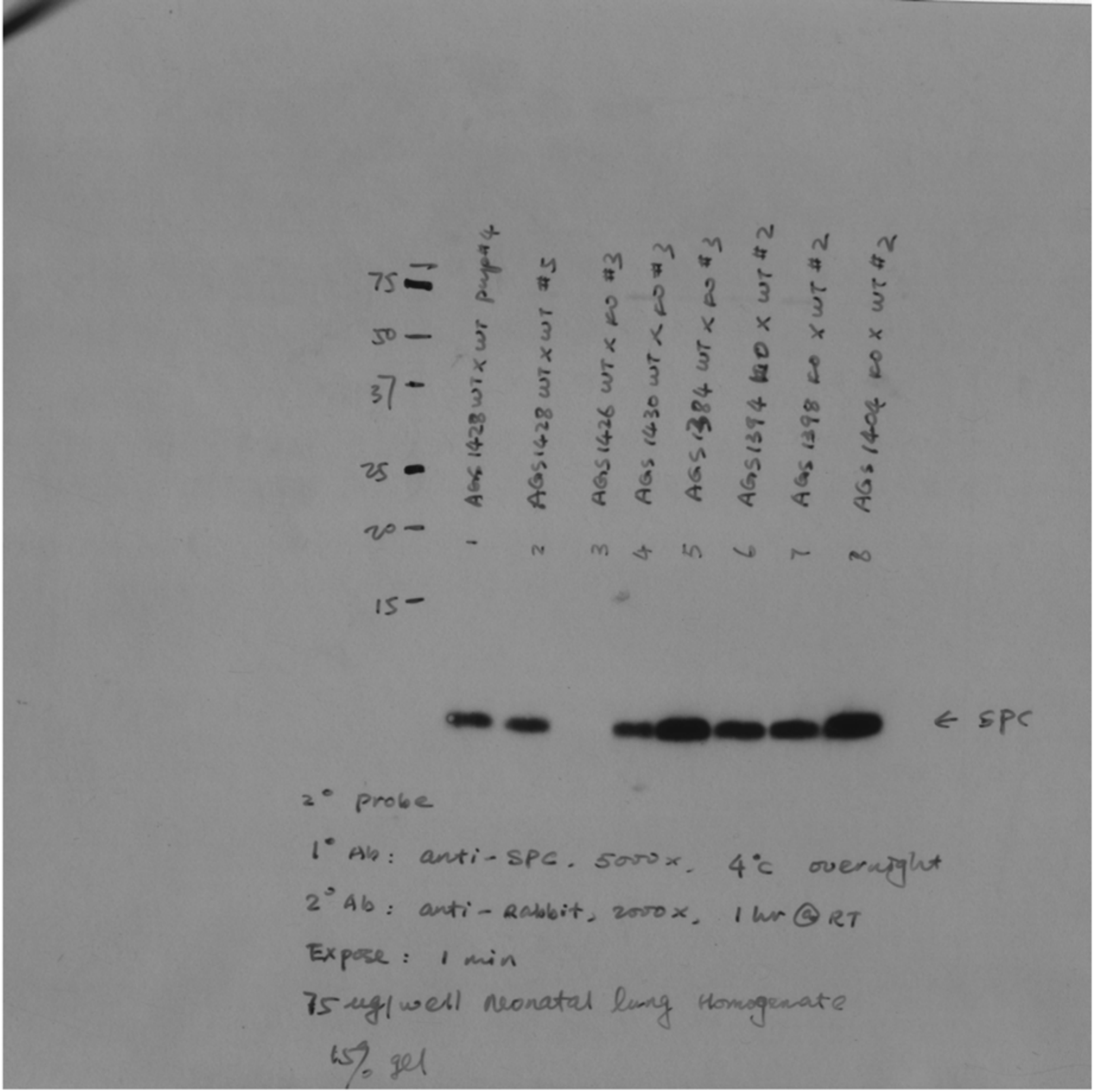

75
50
37
25
20
15
1 AGS 1428 WT x WT pup #4
2 AGS 1428 WT x WT #5
3 AGS 1426 WT x KO #3
4 AGS 1430 WT x KO #3
5 AGS 1384 WT x KO #3
6 AGS 1394 KO x WT #2
7 AGS 1398 KO x WT #2
8 AGS 1404 KO x WT #2
← SPC
2° probe
1° Ab: anti-SPC, 5000x, 4°C overnight
2° Ab: anti-Rabbit, 2000x, 1 hr @ RT
Expose: 1 min
75 ug/well neonatal lung homogenate
15% gel

SftPC – Lung

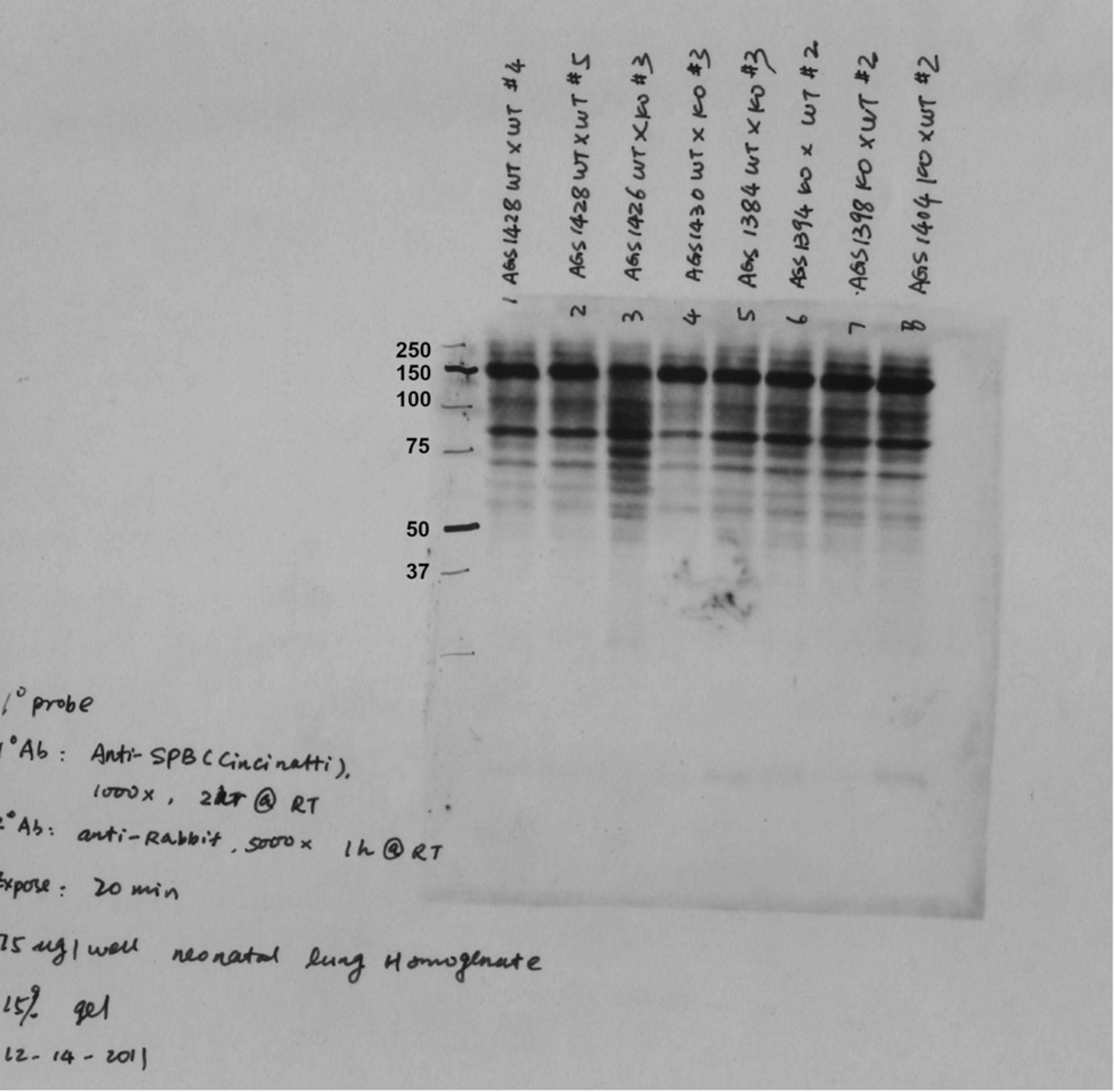

1 AGS1428 WT x WT #4
2 AGS1428 WT x WT #5
3 AGS1426 WT x KO #3
4 AGS1430 WT x KO #3
5 AGS 1384 WT x KO #3
6 AGS1394 KO x WT #2
7 AGS1398 KO x WT #2
8 AGS1404 KO x WT #2
250
150
100
75
50
37
1° probe
1°Ab: Anti-SPB (Cincinatti),
1000x, 2hr @ RT
2°Ab: anti-Rabbit, 5000x 1h @ RT
Expose: 20 min
25 ug/well neonatal lung Homoglnate
15% gel
12-14-2011

SPB – Lung
